# *Vaccinium* spp. Berries in the Prevention and Treatment of Non-Alcoholic Fatty Liver Disease: A Comprehensive Update of Preclinical and Clinical Research

**DOI:** 10.3390/nu16172940

**Published:** 2024-09-02

**Authors:** Ewelina Książek, Zuzanna Goluch, Marta Bochniak

**Affiliations:** 1 Department of Agroenginieering and Quality Analysis, Faculty of Production Engineering, Wroclaw University of Economics and Business, Komandorska 118-120, 53-345 Wrocław, Poland; marta.bochniak@ue.wroc.pl; 2 Department of Food Technology and Nutrition, Faculty of Production Engineering, Wroclaw University of Economics and Business, Komandorska 118-120, 53-345 Wrocław, Poland; zuzanna.goluch@ue.wroc.pl

**Keywords:** non-alcoholic fatty liver disease, NAFLD, NASH, liver steatosis, *Vaccinium* spp., blueberry, bilberry, PRISMA, fruits, juices, extract, supplement, animal model, cell line

## Abstract

Non-alcoholic fatty liver disease (NAFLD) is a common chronic liver disorder marked by the buildup of triacylglycerols (TGs) in the liver. It includes a range of conditions, from simple steatosis to more severe forms like non-alcoholic steatohepatitis (NASH), which can advance to fibrosis, cirrhosis, and hepatocellular carcinoma. NAFLD’s prevalence is rising globally, estimated between 10% and 50%. The disease is linked to comorbidities such as obesity, type 2 diabetes, insulin resistance, and cardiovascular diseases and currently lacks effective treatment options. Therefore, researchers are focusing on evaluating the impact of adjunctive herbal therapies in individuals with NAFLD. One herbal therapy showing positive results in animal models and clinical studies is fruits from the *Vaccinium* spp. genus. This review presents an overview of the association between consuming fruits, juices, and extracts from *Vaccinium* spp. and NAFLD. The search used the following keywords: ((*Vaccinium* OR blueberry OR bilberry OR cranberry) AND (“non-alcoholic fatty liver disease” OR “non-alcoholic steatohepatitis”)). Exclusion criteria included reviews, research notes, book chapters, case studies, and grants. The review included 20 studies: 2 clinical trials and 18 studies on animals and cell lines. The findings indicate that juices and extracts from *Vaccinium* fruits and leaves have significant potential in addressing NAFLD by improving lipid and glucose metabolism and boosting antioxidant and anti-inflammatory responses. In conclusion, blueberries appear to have the potential to alleviate NAFLD, but more clinical trials are needed to confirm these benefits.

## 1. Introduction

Non-alcoholic fatty liver disease (NAFLD) is a prevalent and escalating health concern with significant implications for liver-related morbidity and mortality [1]. This condition includes a range of metabolic disorders associated with insulin resistance (IR), progressing from simple fatty liver (NAFL) to more severe forms such as non-alcoholic steatohepatitis (NASH), cirrhosis, and hepatocellular carcinoma (HCC) [2,3,4]. The prevalence of NAFLD in the general population worldwide varies from 9% to 30%, with the highest rates observed in industrialized communities [5,6]. In Europe, the prevalence of NAFLD reaches 25% [1]. The prevalence is higher among men (40%) than women (26%). It is projected that the prevalence of NAFLD will significantly increase in many world regions by 2030 [7].

NAFLD is induced by increased accumulation of triacylglycerols (TGs), leading to hepatic steatosis, excluding alcohol abuse as a pathogenic factor, although with a histopathological picture resembling alcohol-induced liver damage [8]. The pathogenesis of NAFLD is multifactorial and involves factors such as insulin resistance, oxidative stress, inflammation, and mitochondrial dysfunction [9,10]. Furthermore, the gut–liver axis and gut microbiota have been linked to the progression of NAFLD by influencing gut homeostasis [9,11]. In NAFLD, disrupted insulin signaling and heightened lipid accumulation are key contributors to hepatocyte injury, leading to increased levels of liver enzymes such as aspartate aminotransferase (AST) and alanine aminotransferase (ALT), which serve as serum indicators of liver damage [12].

The disease often progresses asymptotically in its early stages, which poses challenges for early detection and intervention. NAFLD has emerged as the most prevalent form of chronic liver disease globally, concurrent with the rise in obesity and type 2 diabetes. It is associated with an elevated risk of cardiovascular complications, underscoring its impact on overall health [13]. Additionally, NAFLD is linked to diminished quality of life and heightened mortality risk, with cardiovascular disease being the primary cause of mortality, followed by extrahepatic cancer, liver-related mortality, and diabetes [14,15,16]. NAFLD represents a complex and multifaceted disease with profound implications extending beyond liver health. Understanding the diverse etiological factors contributing to NAFLD, including metabolic, inflammatory, and genetic factors, is pivotal for developing effective prevention and treatment strategies for this increasingly prevalent disorder.

Multiple pharmacological approaches have been explored in the treatment of NAFLD. Current drug therapies targeting comorbidities associated with metabolic syndrome primarily involve combinations of various agents. These treatment approaches include antioxidants like vitamins E and C and betaine; insulin sensitizers such as thiazolidinediones and metformin; lipid-lowering medications like statins, orlistat, and probucol; cytoprotective agents like ursodeoxycholic acid; and anti-inflammatory or antifibrotic drugs, including pentoxifylline and angiotensin receptor blockers [8,17]. The threat of NAFLD to human health is gradually increasing, underscoring the need for continued research into potential therapeutic targets. Numerous studies indicate that patients with NAFLD suffer from various metabolic disorders, such as dyslipidemia and glucose regulation disturbances, which further exacerbate the condition. The close relationship between metabolism and NAFLD highlights the necessity of metabolic therapy. It is worth considering the use of drugs targeting related diseases in combination with those aimed at NAFLD. Although many compounds have shown promising potential in preclinical studies, their outcomes in clinical trials remain unsatisfactory [18,19,20]. Despite the registration of over 1400 clinical trials as of August 2024 (https://clinicaltrials.gov/), progress in the treatment of NAFLD and NASH remains limited. The first drug approved by the Food and Drug Administration (FDA) in March 2024 is resmetirom, which was authorized for use in the United States under an accelerated approval process in combination with diet and exercise for the treatment of adults with NASH and moderate to advanced liver fibrosis (stages F2 to F3). In the European Union, resmetirom is currently under regulatory review for treating NASH. Resmetirom also reduced NAFLD activity by ≥2 points and improved fibrosis by at least one stage without worsening the NAFLD activity score. Resmetirom is an oral THR-β agonist. Hepatotoxicity and adverse effects, such as diarrhea, pruritus, and nausea, have been observed during resmetirom treatment [21,22].

Dietary interventions and physical activity (PA) are broadly recognized as cornerstones in the management of NAFLD/NASH [23]. In routine clinical practice, maintaining a healthy weight, adhering to a well-balanced diet with adequate energy intake, and engaging in physical activity or resistance training are key strategies for preventing and treating NAFLD [24]. Current AASLD, EASL, and ESPEN guidelines recommend weight loss through hypocaloric diets combined with increased physical activity as a primary approach to managing NAFLD/NASH [25]. Although current treatment methods combined with lifestyle changes have proven effective for NAFLD, patient adherence to recommendations is often inadequate. As a result, there is an urgent need to develop innovative therapies that provide high efficacy with minimal side effects for the treatment of NAFLD.

In recent years, natural products have played a significant role in drug development and design, as an increasing number of them demonstrate considerable therapeutic potential in treating various diseases, including NAFLD [25]. Progress in the development of natural products for NAFLD is evident in clinical research, with 63 out of 1407 studies focusing on natural products (August 2024, according to https://clinicaltrials.gov/). To date, studies have shown that berberine and silymarin improve lipid accumulation in the liver of patients and are currently in phase IV clinical trials, potentially leading to new treatments for NAFLD [26,27,28].

Compounds such as flavonoids, polyphenols, and phytochemicals present in fruits, vegetables, and medicinal plants have exhibited promising therapeutic effects in NAFLD, including attenuation of liver enzymes, antioxidant properties, and anti-inflammatory actions [29]. Plant-derived foods rich in bioactive compounds represent potential natural interventions for preventing and mitigating fatty liver disease [30]. Consequently, researchers are evaluating the impact of adjunctive herbal therapies on individuals with NAFLD [8,30]. One such herbal therapy demonstrating favorable outcomes in preclinical and clinical studies is *Vaccinium* spp. blueberry. Blueberry supplementation has been shown to mitigate hepatic lipid accumulation, oxidative stress, and inflammatory responses by modulating Notch1 signaling, a key regulator of liver lipid metabolism [31,32]. Similarly, cranberry supplementation has been associated with ameliorating high-fat-diet-induced NAFLD in murine models, attributed to its anti-inflammatory and antioxidant properties [33]. Notably, a study by Hormoznejad et al. observed a significant reduction in fibrosis severity among NAFLD patients receiving cranberry supplementation compared to placebo [34]. Current knowledge regarding the full utilization of *Vaccinium* fruits and leaves remains limited, and conclusive clinical results have not yet been achieved. Therefore, significant efforts are still needed to thoroughly elucidate the mechanisms and targets of these compounds, including their hepatoprotective effects, and to develop research strategies that will provide a foundation for creating new drugs.

Studies indicate that blueberry consumption does not lead to significant side effects, making it safe for daily intake and beneficial to health [35,36]. The extract from *Vaccinium arctostaphylos* berries has been shown to be safe, with no adverse effects on the hematopoietic system, liver, or kidneys and no undesirable interactions in patients [37]. Similarly, extracts from *Vaccinium myrtillus* have been well-tolerated in toxicity studies on dogs and rodents, where no adverse effects were reported [38].

Due to the prevalence of NAFLD and the lack of effective treatment modalities, we hypothesized that blueberry supplementation could effectively mitigate the severity of hepatic steatosis in NAFLD patients. To elucidate the mechanisms of action of *Vaccinium* spp. fruits in NAFLD and their therapeutic potential, a comprehensive literature review encompassing in vitro, in vivo, and clinical studies was conducted. This review aims to present an overview of the relationship between the consumption of Vaccinium spp. fruits—including juices and extracts—and NAFLD in the context of current achievements.

## 2. Materials and Methods

### 2.1. Search Strategy

The current systematic review was conducted following the Preferred Reporting Items for Systematic Reviews and Meta-Analyses (PRISMA) guidelines [39]. Electronic searches were performed in the academic libraries of Cochrane, Ebsco, PubMed, Embase, Scopus, and Web of Science. The searches combined MeSH terms and keywords, utilizing quotation marks and field tags with Boolean operators. The following keywords were employed for the search: ((*Vaccinium* OR blueberry OR bilberry OR cranberry) AND (“non-alcoholic fatty liver disease” OR “non-alcoholic steatohepatitis”)).

Furthermore, to ensure the comprehensiveness of the searches, references of included studies were examined for additional potential sources.

### 2.2. Selection Criteria

For all databases, the fundamental stages of exclusion were determined as follows: 1. keyword search; 2. publication years (2011–2024); 3. language (English); 4. publication type (article). Selection occurred across the following domains: title, abstract, keywords (Cochrane, Ebsco, Embase, PubMed, Scopus), and topic (Web of Science). The search and selection process engaged three independent researchers operating concurrently. Results from each database were exported to files in CSV or Excel format, and summaries containing publication information, including abstracts, were generated. Exclusion criteria comprised reviews, research notes, book chapters, case studies, and grants. Three independent researchers scrutinized titles and abstracts. Articles failing to meet inclusion criteria underwent exclusion through deliberation. Two independently operating researchers analyzed the acquired results to mitigate errors. Any discrepancies were resolved through discussion.

Selected studies adhered to the following criteria: (a) experimental investigations involving cellular models, animals, and randomized controlled trials (RCTs); (b) evaluation of the effects of extracts, beverages, powders, or juices on hepatic enzyme levels; (c) provision of adequate data about hepatic enzyme levels.

### 2.3. Methodological Quality Assessment

Three researchers independently reviewed the methodology of the included animal studies using the Stroke Therapy Academic Industry Roundtable guidelines [40] and assessed clinical trials with the Jadad scale [41]. The researchers evaluated the quality of each animal study based on criteria such as sample size calculation, inclusion/exclusion criteria, randomization, allocation concealment, exclusion reporting, blinded outcome assessment, and disclosure of conflicts of interest and funding. The Jadad scale rated clinical trials on randomization (2 points), double blinding (2 points), and withdrawals/dropouts (1 point), with scores above 4 indicating high quality.

### 2.4. Study Selection

Given the rapidly evolving number and diversity of databases, information retrieval specialists trained in systematic literature searching were engaged to ensure the high quality of the search process. Each team member independently conducted systematic searches to identify as many eligible studies as possible. To ensure the reliability of the findings, the abstracts were reviewed independently and in duplicate. Following discussions, the researchers decided to include preclinical studies in addition to randomized controlled trials (RCTs) due to the minimal number of clinical studies available on the use of Vaccinium berries in the alleviation of NAFLD.

The team opted not to perform a meta-analysis for three reasons: (1) the existing clinical literature was insufficient, with only two studies available; (2) the included studies differed significantly in terms of dosage, duration, sample size, and method of administration of the active substance; and (3) the review focused on processes, theory development, and a qualitative description of the studies. Data analysis and quality assessment of the articles were conducted independently by three researchers, with the results of each study recorded in an evidence table.

**Figure 1:**
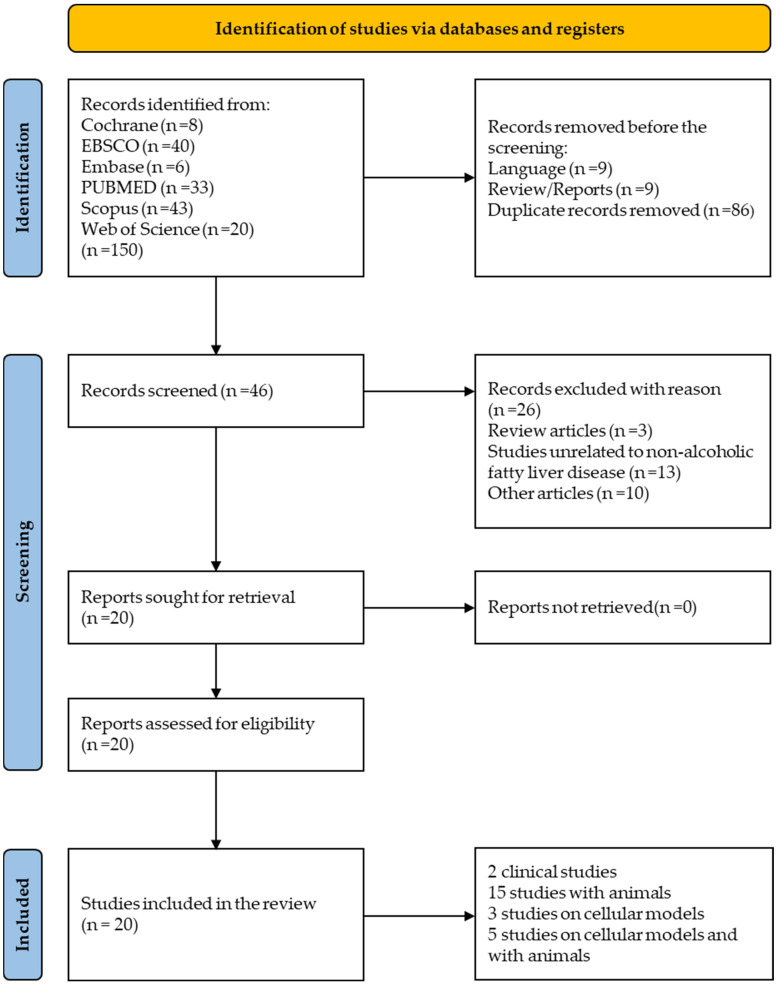
Flow diagram of the study selection process.

The article selection process was carried out in three stages. In the first stage, three researchers initially screened titles and abstracts. In the second stage, a detailed assessment of the articles was performed based on predefined selection criteria, developed according to the research question, with the results organized in a table. In the third stage, one researcher integrated all results into a single document, and the articles were subjected to detailed analysis to determine their inclusion in the study. The selected studies were then organized by year of publication and alphabetically by the first author’s surname. Any disagreements were discussed and resolved through consultation among the researchers. The preliminary searches produced 150 results, and after removing duplicates, 46 peer-reviewed articles were selected for further consideration based on relevance (Figure 1).

## 3. Results

### 3.1. Overview of Vaccinium spp.

The *Vaccinium* species, belonging to the *Ericaceae* family and the *Rhododendron* genus, complement the morphologically dominant taxon, which encompasses 4250 species. In Europe, the species of this genus include *Vaccinium myrtillus* L., *Vaccinium vitis-idaea* L., *Vaccinium oxycoccus* L., and *Vaccinium uliginosum* L. The flowers, leaves, and fruits of these plants are widely used in traditional medicine [42]. Due to their numerous health benefits, these plants are utilized in the prevention and treatment of various conditions, including cardiovascular diseases, neurodegenerative disorders, infections, rheumatoid arthritis, and cancer [43,44,45,46,47,48]. Their primary health effects are attributed to their antioxidant, antimicrobial, and detoxifying properties. It is also noteworthy that these plants, mainly those rich in polyphenols such as anthocyanins, exhibit a particularly beneficial impact on health [49,50]. Anthocyanins are the primary compounds isolated and identified in berries, constituting 0.1 to 0.25% of the fresh fruit weight, as well as in leaves, alongside other active components such as resveratrol, flavonols (e.g., quercetin, catechins), phenolic acids, ellagitannins, and iridoids [42]. The presence of various types of anthocyanins characterizes the fruits of the Vaccinium genus. Specifically, compounds such as cyanidin 3-O-galactoside, cyanidin 3-O-glucoside, cyanidin 3-O-arabinoside, delphinidin 3-O-galactoside, delphinidin 3-O-arabinoside, delphinidin 3-O-glucoside, malvidin 3-O-galactoside, malvidin 3-O-arabinoside, malvidin 3-O-glucoside, petunidin 3-O-galactoside, petunidin 3-O-arabinoside, petunidin 3-O-acetylglucoside, peonidin 3-O-galactoside, and peonidin 3-O-arabinoside have been identified [51,52].

Studies suggest that anthocyanins may have a beneficial impact on liver health, particularly in the context of NAFLD, by balancing lipid storage and metabolism. Incubation of HepG2 cells, treated with oleic acid, with petunidin-3-O-galactoside (100 μM), petunidin-3-O-glucoside (50 and 100 μM), and malvidin-3-O-galactoside (100 μM) derived from *Vaccinium virgatum* fruits for 12 h significantly reduced intracellular TC and TG levels compared to the model group. Malvidin and petunidin exhibited more significant hypolipidemic activity than delphinidins in HepG2 cells [53]. Among the anthocyanins present in blueberries, malvidin-3-O-glucoside and malvidin-3-O-galactoside significantly alleviated FFA-induced lipid accumulation. Additionally, malvidin-3-O-glucoside (M3G) and malvidin-3-O-galactoside inhibited oxidative stress by suppressing ROS, increasing glutathione levels, and enhancing antioxidant enzyme activity [54]. The study on the effects of malvidin, malvidin-3-glucoside, and malvidin-3-galactoside from *Vaccinium ashei* in the human HepG2 cell line and in streptozotocin-induced diabetic mice demonstrated that anthocyanins reduced ROS levels by 80%, 76%, and 91% and increased HepG2 cell viability by 79%, 73%, and 98%, respectively. They also inhibited hyperglycemia and hyperlipidemia by decreasing the expression levels of enzymes involved in gluconeogenesis and lipogenesis and increasing those engaged in glycogenolysis and lipolysis via the AMPK signaling pathway in HepG2 cells [55].

Anthocyanins may exhibit potential in alleviating NAFLD due to their antioxidant properties and effectiveness in controlling lipid metabolism, glucose homeostasis, and transcription factors [56]. Unfortunately, these compounds are highly susceptible to degradation when exposed to high pH, light, heat, and oxygen during processing and storage and to interactions with other food components and additives, leading to poor bioavailability and reduced bioactivity [49]. Anthocyanins typically exist in the form of a flavylium cation, which predominates in highly acidic aqueous solutions (pH < 2). Under the pH conditions found in plants, food, and the gastrointestinal tract (from pH 2 to pH 8), anthocyanins transform into a mixture of colored and colorless forms in equilibrium through acid–base reactions, water addition–elimination processes, and isomerization. Each chemical species is characterized by specific features (such as charge, electron distribution, planarity, and shape), which affect its reactivity and interactions with plant or food components, including other phenolic compounds [57]. It is believed that the bioavailability of anthocyanins is among the lowest of all flavonoids, amounting to only 1–2% [58]. However, studies by Czank K. et al. [59] report that anthocyanins have a minimal relative bioavailability of 12.3 ± 1.3%, with their metabolites reaching peak serum concentrations 42 times higher. Anthocyanins are typically consumed as a mixture of various compounds and structural forms, which can undergo further transformation, metabolism, and degradation in different sections of the gastrointestinal tract and other body compartments. Human intervention studies on absorption, distribution, metabolism, and elimination suggest that peak concentrations of anthocyanins and phase II anthocyanidin conjugates in serum are reached after approximately 1.5 h, with levels around 100 nmol/l following doses of ≤500 mg of anthocyanins. This indicates anthocyanins are significantly less bioavailable than other flavonoids [58,59,60]. Anthocyanins are likely absorbed across the intestinal membrane via transporters such as OATP 2B1 (for all forms), GLUT2 (for both aglycones and glycosylated forms), and SGLT1 (for glycosylated forms), though these absorption pathways require further in-depth validation through research. Given the diversity of anthocyanins in nature, depending on the aglycone structures and types of sugar molecules, it is essential to conduct additional studies on structure–activity relationships to understand better their bioavailability in the human body [61,62].

Low bioavailability, primarily due to limited stability, is a significant drawback associated with anthocyanins. Therefore, preserving these compounds present in blueberries and enhancing their bioavailability are crucial [61]. Consequently, efforts are being made to enhance the limited bioavailability of phytochemicals present in blueberries. These strategies include the use of various delivery systems such as encapsulation in lipid nanocarriers or liposomes, emulsions, micelles, incorporation into polymeric nanoparticles, solid dispersions, and nanocrystals [63]. In recent years, research has focused on nanoencapsulation, microencapsulation, and protein complexes. Macromolecular components are currently considered effective encapsulating carriers that protect anthocyanins from degradation during digestion [61]. Studies by Lang X. et al. [64] have demonstrated that the two main casein monomers, α-casein and β-casein, can preserve the stability and antioxidant capacity of blueberry anthocyanins under processing conditions. Further research by Lang X. et al. [65] showed that both α-casein and β-casein can enhance the stability of blueberry anthocyanins during intestinal digestion and protect their antioxidant properties. Additionally, the inclusion of α-casein or β-casein could improve the bioavailability of blueberry anthocyanins. To enhance the bioavailability and applications of anthocyanins, nanocomplexes have also been employed, which may exert a protective effect on the gastrointestinal tract and other processes. Ge et al. observed that anthocyanin nanocomplexes, prepared from chitosan hydrochloride and carboxymethyl chitosan, exhibited improved stability when stored at various temperatures and pH levels. The bioavailability of anthocyanins in these nanocomplexes reached 40.1%, compared to 17.2% for free anthocyanins, indicating a significant improvement in bioavailability due to encapsulation in nanocomplexes [66,67]. In contrast, nanoliposomes containing lecithin and cholesterol, prepared using enhanced supercritical carbon dioxide, provided additional stability to anthocyanins during storage and simulated gastrointestinal digestion. Protection of anthocyanins was observed during simulated intestinal digestion, where encapsulated anthocyanins decreased to 72.76% compared to 52.01% for free anthocyanins. Unfortunately, the use of nanoliposomes as protectors for anthocyanins appears to be limited by their instability when in contact with pancreatin [68]. Microencapsulation effectively stabilizes anthocyanins against degradation caused by light and oxygen, enhancing their utility in functional products [69]. Encapsulation using a combination of carboxymethyl starch and xanthan gum has demonstrated enhanced thermal stability of blueberry anthocyanins. Additionally, storage stability results showed that the stability of anthocyanins increased to 76.11% after 30 days of storage at 37 °C. The anthocyanins were also primarily retained within the microcapsules in the stomach and released in the intestine [70]. Nanoparticle systems, microcapsules, and protein complexes contribute to enhancing the stability and bioavailability of anthocyanins. However, the results of research and technological innovations require further application to develop safe products that provide health benefits to consumers.

### 3.2. Study Selection and Characteristics

A comprehensive search across multiple databases, including Cochrane, Ebsco, Embase, PubMed, Scopus, and Web of Science, yielded 150 articles. After removing duplicates, 64 articles remained. Of these, 18 were excluded due to being in a language other than English or not being original research. An additional 18 articles were excluded because they did not pertain to NAFLD or failed to meet the inclusion criteria. Finally, 20 articles met the inclusion criteria and were included in the analysis. The characteristics of the included studies are presented in Table 1, Table 2 and Table 3. All studies were published between 2011 and 2024. The selected articles included clinical studies (2), animal studies (15), cell line studies (3), and studies involving both animal and cell line research (5).

In the clinical studies, the Kolmogorov–Smirnov test was used to assess data distribution. For comparing variables within groups, either the paired *t*-test or the Wilcoxon test was employed, while for comparing variables between two groups, either the independent *t*-test or the Mann–Whitney U test was used. A significance level of *p* < 0.05 was applied in the studies. In the studies using cellular and animal models, statistical differences in data were assessed using the independent samples *t*-test and one-way analysis of variance (ANOVA) [31,32,33,35,71,72,73,74,75,76,77,78,79,80], with post hoc comparisons performed using Duncan’s multiple-range tests [81]. Multiple comparisons were also conducted using Tukey’s test for parametric variables and the Kruskal–Wallis test for non-parametric variables [72,82,83]. In one study, the statistical significance of differences was determined using Tukey’s test [84]. In the study by Sotelo-Gonzales et al. [82], principal component analysis (PCA), sparse partial least squares discriminant analysis (sPLS-DA), and K-means clustering were also performed based on the urinary metabolite profiles of each experimental group. Statistical differences at *p* < 0.05 were considered significant in all the cited studies.

### 3.3. Clinical Study

Clinical studies evaluating the effects of plants from the genus *Vaccinium* are limited. There are only two studies assessing the impact of cranberry tablets on patients with NAFLD, which are summarized in Table 1 [34,85]. Both human studies are randomized controlled trials conducted directly on patients with NAFLD, diagnosed based on liver ultrasonography. Pregnant and breastfeeding individuals, as well as those with diabetes, other liver diseases, heart failure, renal insufficiency, pulmonary insufficiency, alcohol abuse, and those using antioxidant or vitamin supplements other than vitamin E were excluded from both studies. The duration of the trials ranged from 12 weeks and 6 months.

In the study conducted by Hormoznejad R. et al. [34], patients with NAFLD (18 females and 23 males) were randomly allocated to receive either a cranberry supplement or a placebo for 12 weeks, administered twice daily. Participants in the cranberry group received tablets containing 144 mg of *Vaccinium macrocarpon* extract, providing at least 36 mg of proanthocyanidins, equivalent to 13 g of dried cranberry fruit. The remaining composition of the tablets was unspecified. Placebo tablets contained 288 mg of starch. All patients followed a hypocaloric diet, consuming 500–1000 kcal less than their estimated energy needs. Fasting insulin levels significantly decreased in both groups; however, the reduction was more significant in the cranberry group compared to the placebo group (from 10.55 to 8.20 µ/mL and from 10.66 to 9.80 µ/mL, respectively, *p* = 0.005). The homeostatic model assessment of insulin resistance (HOMA-IR) index showed significant improvement in the cranberry group, decreasing to 1.88. No significant differences between the cranberry and placebo groups were observed in the TG, TC, and LDL-C levels (*p* > 0.05). Significant improvements in ALT levels were noted in both groups posttreatment compared to baseline, with a markedly more significant reduction in the cranberry group compared to the placebo group (from 58.35 to 36.90 IU/L and from 55.33 to 45.42 IU/L, respectively; *p* = 0.040). Ultrasound assessments revealed a significant decrease in liver steatosis in both groups after the intervention (*p* < 0.001), with no significant differences between the postintervention data of the two groups.

In the study by Shirazi M.K. et al. [85], 110 patients diagnosed with NAFLD were initially enrolled, with data ultimately analyzed from 94 participants (49 females, 45 males) due to dropouts from nine in the intervention group and seven in the control group. The diagnosis was confirmed via liver ultrasound by gastroenterology specialists. The study included adults over 18 who followed a hypocaloric diet, reduced their daily intake by 500 kcal below their estimated energy needs, and received vitamin E supplementation. The intervention group, comprising 46 patients, was given one cranberry capsule (144 mg) daily, while the placebo group, consisting of 48 patients, received a placebo with the same base formula minus the active ingredient for a duration of six months. The cranberry capsules contained 144 mg of *Vaccinium macrocarpon*, equivalent to 13 g of dried cranberry fruit. Out of one hundred and ten patients with NAFLD, nine from the intervention group and seven from the control group did not continue with the observation. In lipid profile measurements, average levels of TC (189.02 mmol/L; *p* < 0.001) and TG (190.54 mmol/L; *p* = 0.01) were significantly lower in the cranberry group compared to the placebo group (200.29 mmol/L and 188.54 mmol/L, respectively). At the end of the intervention, average levels of insulin and HOMA-IR were significantly lower in the cranberry group (5.62 μL/mL and 1.39, respectively) compared to the placebo group (10.06 μL/mL and 2.51, respectively).

**Table 1:** Characteristics of the included randomized clinical trials regarding the impact of *Vaccinium macrocarpon* on liver enzyme levels.

Study	Study Design	Study Population	Type of Intervention	Dose (mg/day)	Trial Duration	ALT Levels (U/L)	AST Levels (U/L)	Insulin (µ/mL)	HOMA IR	Jadad Scale
Hormoznejad et al. 2020 [34]	Randomized double-blind, placebo-controlled clinical trial	Age ≥ 18 years; BMI 25 ± 5 kg/m^2^, N = 41 (groups: cranberry n = 20, placebo n = 21)	The placebo and cranberry groups received either placebo or cranberry tablets (two tablets; one tablet after lunch and another one after dinner)	288	12 weeks	Before: in the cranberry group: 58.35 ± 18.03; in the placebo group: 55.33 ± 26.10; after: in the cranberry group: 36.90 ± 9.00; in the placebo group: 45.42 ± 15.59	Before: in the cranberry group: 26.85 ± 10.30; in the placebo group: 29.95 ± 15.02; after: in the cranberry group: 22.60 ± 7.68; in the placebo group: 24.90 ± 15.79	Before: in the cranberry group: 10.55 ± 1.43; in the placebo group: 10.66 ± 1.55; after: in the cranberry group: 8.20 ± 0.61; in the placebo group: 9.80 ± 1.36	Before: in the cranberry group: 2.59 ± 0.86; in the placebo group: 2.38 ± 0.71; after: in the cranberry group: 1.88 ± 0.20; in the placebo group: 2.20 ± 0.45	5
Shirazi et al. 2021 [85]	Randomized double-blind, placebo-controlled clinical trial (parallel)	Age ≥ 18 years, N = 110 (groups: cranberry n = 46, placebo n = 48)	The cranberry capsule includes 144 mg *Vaccinium macrocarpon* (equal to 13 g dried cranberry fruit)	144	6 months	Before: in the cranberry group: 42.74 ± 15.04; in the placebo group: 47.48 ± 18.35; after: in the cranberry group: 39.54 ± 16.95; in the placebo group: 38.69 ± 14.20	Before: in the cranberry group: 37.22 ± 13.51; in the placebo group: 41.17 ± 16.69; after: in the cranberry group: 32.98 ± 14.33; in the placebo group: 31.98 ± 12.48	Before: in the cranberry group: 10.38 ± 3.09; in the placebo group: 10.65 ± 3.02; after: in the cranberry group: 5.62 ± 2.04; in the placebo group: 10.06 ± 2.94	Before: in the cranberry group: 2.78 ± 0.99; in the placebo group: 2.84 ± 0.98; after: in the cranberry group: 1.39 ± 0.62; in the placebo group: 2.51 ± 0.85	5

ALT—alanine aminotransferase; AST—aspartate transaminase; HOMA-IR—homeostatic model assessment for insulin resistance.

### 3.4. Preclinical Study

Preclinical studies evaluated the effects of standardized aqueous or alcoholic extracts [31,32,74,82], juices [75,76], powders [33,35,80,84], nutraceuticals [71,72,81], and preparations containing monomers [77,78] from berry fruits in various animal models, primarily in male Sprague Dawley rats and male C57BL/6N mice. The animal models in these studies were categorized into (1) dietary supplementation, (2) pharmacological intervention, (3) genetic mutation, or (4) a combination of these approaches. Various diets were employed to simulate NAFLD or NASH, including Western, high-fat, and high-cholesterol diets, all containing elevated sucrose, fructose, soybean oil, or lard levels. These dietary regimes resulted in liver alterations, weight gain, and insulin resistance, critical preclinical research issues. The summary of preclinical studies is presented in Table 2.

#### 3.4.1. *Vaccinium* and the Alleviation of Liver Steatosis and Hepatocellular Damage

In the analyzed studies, the use of extracts or juices from Vaccinium berries in combination with diet-induced NAFLD in animal models was investigated. In the studies by Ren et al. [75], the administration of berry juice to Sprague Dawley rats (200 to 250 g) at a dose of 15 g/kg body weight once daily contributed to mitigating the severity of NAFLD and lipid degeneration of hepatocytes. In the control group of the NAFLD model, hepatocyte lobular structures were disrupted, and hepatic plates were scattered with numerous lipid droplets. The disrupted liver structures were effectively restored and organized around the central vein in the berry juice experimental model. Several small vacuoles and numerous small red lipid droplets were also observed. In the study by Morrison et al. [72], administering Mirtoselect at a 0.1% (*w*/*w*) concentration helped slow down the progression of liver steatosis caused by a Western-type diet, which includes 15% cocoa butter, 1% corn oil, 40.5% sucrose, 20% acid casein, 10% corn starch, and 6.2% cellulose. The treatment significantly reduced macrovesicular steatosis (*p* < 0.001) and also decreased microvesicular steatosis (*p* = 0.027). Histological examination of the mice indicated fewer enlarged hepatocytes lacking cytokeratin 18, indicating a protective effect against liver cell damage. In research conducted by Glisan et al. [33], dietary supplementation with blueberry extract led to a 31.2% reduction in plasma ALT levels in obese mice on a high-fat diet compared to those on a high-fat diet alone. Although the total number of hepatic lipid droplets did not significantly differ between the groups, histological analysis showed a notable decrease in both the total lipid droplet area and the overall hepatic lipid area in the livers of mice that received the blueberry extract. In the studies by Ren et al. [76], hepatocytes in healthy Sprague Dawley rats regularly surrounded the tubular section, with all lipid droplets being small. In the control group of the NAFLD model, liver cell structures were destroyed and widely dispersed with numerous lipid droplets. The administration of berry juice at a dose of 10 mL/kg body weight of rats reduced the size of lipid droplets. Liver cords exhibited a radial arrangement around the central vein, accompanied by a decrease in the number of lipid droplets. Flow cytometry analysis demonstrated that treatment with berry juice decreased apoptosis rates in NASH models. In the study by Shimizu et al. [84], serum ALT levels in mice fed a high-fat diet supplemented with cranberry and those fed a high-fat diet did not show statistically significant differences. However, ALT levels were lower in mice supplemented with 1% cranberry powder. The same study observed a reduction in lipid droplets and hepatocyte ballooning in mice fed a cranberry-supplemented diet compared to those on a high-fat diet. Haga et al. [74] explored the impact of 5% and 10% bilberry (*Vaccinium myrtillus* L.) fruit extracts on liver steatosis and damage in mice. Their study found that bilberry extracts significantly reduced liver fat accumulation and triglyceride content, as shown by histological analysis and hepatic TG measurements. While a high-fat, high-cholesterol diet caused mild fibrosis in the liver around the portal area, mice treated with bilberry extracts exhibited significantly less fibrosis. Histological analysis of mice on a high-fat diet revealed substantial lipid droplets, ballooning degeneration, and inflammatory cell infiltration, indicating hepatic steatosis and inflammation. In contrast, liver sections from the group receiving lyophilized bilberry leaf extracts at a dose of 400 mg/kg per day showed very mild steatosis and inflammatory foci, suggesting that high doses effectively alleviated diet-induced hepatic steatosis and inflammatory infiltration [35]. The administration of a 2% anthocyanin extract from bilberries to laboratory animals on a Western diet significantly reduced lipid content in the liver compared to both the normal diet and Western diet groups [81]. Faheem et al. [71] observed that liver sections of rats treated exclusively with cranberry showed normal liver architecture. Meanwhile, animals on a high-fat and high-cholesterol diet concurrently treated with cranberry (100 mg/kg) exhibited only mild congestion in the portal vein associated with mild ballooning degeneration in hepatocytes and no signs of fibrosis. Nanoparticles resembling exosomes, isolated from blueberries and administered to mice concurrently with a high-fat diet, demonstrated the ability to reduce lipid droplet accumulation and liver mass. Additionally, qRT-PCR analyses revealed that these exosomes decreased mRNA levels of FAS and ACC1 enzymes in the livers of high-fat-diet-fed mice [78]. In the study conducted by Hewage et al. [31], supplementation with lingonberry significantly reduced hepatic TG accumulation and total cholesterol levels. Additionally, a decreased number and size of vacuoles were observed in liver sections of mice fed a high-fat diet supplemented with lingonberry (5% *w*/*w*). In the study by Zhu et al. [77], supplementation with blueberry monomers (TEC) via gavage at doses of 7.5, 15.0, or 30.0 mg/kg once daily for 6 weeks significantly alleviated hepatic steatosis in mice on a high-fat diet.

#### 3.4.2. *Vaccinium* and the Alleviation of Hepatic Fibrosis

NAFLD encompasses a range of liver damage, from clinically mild intrahepatic fat accumulation (steatosis) to the more advanced non-alcoholic steatohepatitis (NASH), which can progress further to fibrosis, cirrhosis, and hepatocellular carcinoma.

Histological observations by Morrison et al. [72] demonstrated that liver collagen content was significantly lower (*p* = 0.034) in the diet supplemented with Mirtoselect. A significant reduction in Col1a1 expression was also observed compared to the high-cholesterol diet. The expression of the hepatic stellate cell activation marker gene Acta2 (D), as well as the induction of profibrotic cytokines Tgfb1 (E) and Tnf (F), was less pronounced with Mirtoselect supplementation. Mirtoselect strongly inhibited TGF-β signaling activation and suppressed hepatic stellate cell activation. It was concluded that Mirtoselect significantly mitigated disease progression. Faheem et al. [71] observed that cranberry supplementation in the diet reduced TGF-β levels by 28% and 49%, α-SMA levels by 32% and 45%, and hydroxyproline levels by 17% and 37% at doses of 50 mg/kg and 100 mg/kg, respectively. Rats fed a high-fat, high-cholesterol diet and simultaneously treated with cranberry at doses of 50 mg/kg and 100 mg/kg exhibited a significant reduction in collagen deposition. The activation of hepatic stellate cells and liver fibrosis may be driven by the activation of the NLRP3 inflammasome. In the study by Zhu et al. [77], blueberry TEC monomers significantly inhibited pyroptosis and reduced NLRP3 inflammasome activation, suggesting that TEC may improve NASH. Ryyti et al. [80] found at the functional level that lingonberry supplementation prevents the high-fat-diet-induced upregulation of inflammatory response genes, specifically Cxcl14, which is one of the genes involved in liver inflammation and fibrosis.

#### 3.4.3. Anti-Inflammatory Effects of *Vaccinium*

In individuals with NAFLD, lipotoxicity, insulin resistance, and endotoxins trigger the activation of proinflammatory cytokines such as tumor necrosis factor-α (TNF-α), interleukin-1α (IL-1α), interleukin-1β (IL-1β), interleukin-6 (IL-6), and resistin [3]. This inflammation plays a crucial role in the progression from steatosis to NASH. Furthermore, TNF-α and its receptor impede insulin receptors and activate the NF-kB transcription factor [86,87].

A significant reduction in liver inflammation was observed in studies investigating the effects of a high-cholesterol diet combined with the nutraceutical Mirtoselect (*p* < 0.001). Mirtoselect did not influence the expression of Emr1 or Ccl2, indicating its anti-inflammatory effect might limit the influx of other immune cells. Additionally, there was no increase in the hepatic gene expression of the neutrophil marker Mpo, suggesting a decrease in neutrophil infiltration. Supporting this, the expression of the neutrophil chemoattractants Cxcl1 and Cxcl2 was also reduced by the high-cholesterol diet and Mirtoselect combination. Moreover, Mirtoselect mitigated the disease-related increase in hepatic free cholesterol. The high-cholesterol diet significantly enhanced TNF-α and IL-1β signaling, which activated the proinflammatory transcription factor NF-κB. There was a positive correlation between liver-free cholesterol levels and p65-NF-κB transcriptional activation, linking cholesterol to inflammation. Conversely, Mirtoselect helped reduce p65-NF-κB activity [72]. Glisan et al. [33] examined the impact of cranberry extract supplementation on liver inflammation in obese C57BL/6J mice on a high-fat diet. They discovered that the dietary inclusion of cranberry extract lowered the hepatic mRNA expression of TNF-α, Ccl3, and Cox2, as well as the hepatic protein levels of CCL2 and TNF-α when compared to mice on a high-fat diet without the extract. The study also revealed that cranberry extract significantly reduced hepatic mRNA levels of Tlr4 and NF-κB. These findings, alongside the decrease in TNF-α protein levels and the reduction in the liver’s Cox2 and TNF-α mRNA levels, indicate that the extract partially mitigates liver inflammation by influencing the TLR4/NF-κB signaling pathway. Ren et al. [76] investigated the impact of blueberry juice on the development of NASH. In this study, qRT-PCR analysis indicated that mRNA levels of IL-6 and TNF-α were lower with dietary supplementation of blueberry juice in laboratory animals. Treatment with blueberry juice reduced the levels of IL-6 and TNF-α. Similar findings were obtained by Haga et al. [74], who investigated extracts from *Vaccinium myrtillus* L. in a murine model of liver steatosis. The research results demonstrated reduced levels of proinflammatory cytokines TNF-α, IL-9, IL-1β, and IFN-γ. Treatment with cranberry showed a tendency to suppress IL-6 expression, as observed in the study by Shimizu et al. [84]. Cranberry at 50 and 100 mg/kg doses significantly reduced TNF-α levels by 48% and 72%, respectively. Serum levels of IL-6 also significantly decreased by 36% and 73% at doses of 50 and 100 mg/kg, respectively. Cranberry at both doses reduced the activation of NF-κB, which regulates the production of several proinflammatory cytokines, including TNF-α and IL-6 [71]. In the study by Hewage et al. [32], a high-fat diet induced the formation of inflammatory foci in the livers of mice and increased hepatic mRNA levels of IL-6, MCP-1, and TNF-α. Inflammatory foci were not observed in mice supplemented with cranberry. Furthermore, supplementation with cranberry reduced the expression of IL-6, MCP-1, and TNF-α mRNA [80].

Lingonberry supplementation decreased the expression of inflammation-related genes (Saa1, Saa2) that were upregulated by a high-fat diet. In mice, the serum amyloid A (Saa) gene family plays a role in the development of chronic inflammation, fibrosis, and secondary amyloidosis [80].

Recent research involving mice has underscored the importance of hepatocyte inflammasomes in linking early metabolic stress to subsequent hepatocyte death and fibrogenesis in NASH. These inflammasomes, which are multiprotein complexes within the cytoplasm, respond to danger-associated molecular patterns (DAMPs)—such as saturated fatty acids from de novo lipogenesis (DNL)—and pathogen-associated molecular patterns (PAMPs) from gut microbiota that enter the liver via the portal vein. Data indicate that activating inflammasomes in the liver produces proinflammatory cytokines like interleukin (IL)-1β and IL-18 and initiates apoptosis through caspase-1 (Casp1) activation [3,88]. In experiments conducted by Glisan et al. [33], mice given cranberry extract showed a substantial decrease in hepatic expression of NLRP3 (43%) and thioredoxin-interacting protein (Txnip, 30%) compared to control mice fed a high-fat diet. Furthermore, cranberry supplementation resulted in a significant reduction in the expression of PPAR-α, a transcription factor involved in regulating Txnip, and a 35% decrease in hepatic Casp1 expression.

**Figure 2:**
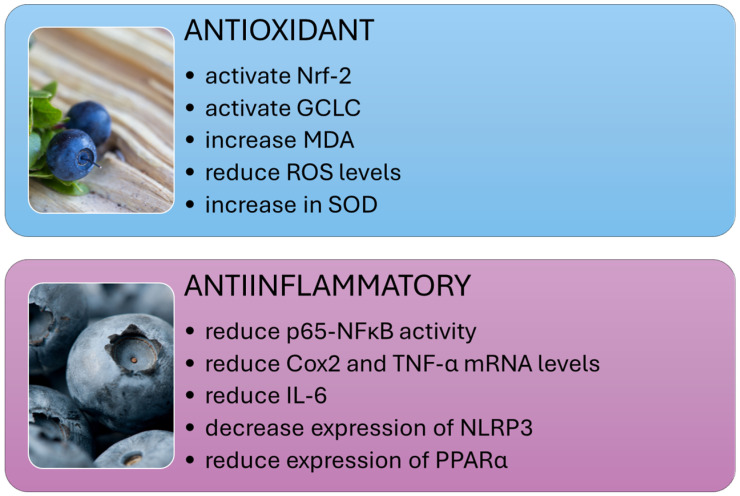
Systemic anti-inflammatory and antioxidant effects of dietary polyphenols.

Studies conducted on various animal models have shown that extracts from cranberry and other plants of the *Vaccinium* genus can significantly reduce levels of proinflammatory cytokines such as TNF-α and IL-6 and modulate signaling pathways, including NF-κB and TLR4 (Figure 2), which are key in the pathogenesis of NAFLD and NASH.

#### 3.4.4. Antioxidant Effects of *Vaccinium*

Elevated free radicals, lipid peroxidation products, and reduced antioxidant levels characterize NAFLD. The accumulation of fat in the liver exacerbates lipotoxicity through high concentrations of free fatty acids (FFAs), free cholesterol, and other lipid metabolites. This leads to oxidative stress, increased reactive oxygen species, and the activation of endoplasmic reticulum stress and mitochondrial dysfunction. The transcription factor nuclear factor-erythroid 2-related factor 2 (Nrf-2) plays a vital role in maintaining cellular redox balance. Research has shown that extracts from *Vaccinium* plants can activate Nrf-2, as well as heme oxygenase-1 (HO-1), NADPH quinone dehydrogenase (NQO1), and glutamate-cysteine ligase catalytic (GCLC) (Figure 2). This activation results in increased production of antioxidant defense enzymes and reduced oxidative stress [56,75,89,90].

For instance, in the livers of high-fat-diet-fed subjects supplemented with *Vaccinium corymbosum* L. leaf extract, a notable increase in the levels of ERRα, Nrf-1, and Nrf-2 genes was observed [35]. Nakano et al. assessed certain antioxidant factors and enzyme levels in the liver. The level of Nrf-2, a key transcription factor regulating the expression of antioxidant proteins, decreased 0.7-fold with a Western diet and returned 1.3-fold with the same diet supplemented with blueberry extract. Additionally, the ubiquitinated Nrf-2 (Ub-Nrf-2) level increased 3.6-fold in animals whose diet included blueberry extracts. Conversely, the level of TBARSs in the liver, a sensitive marker of lipid peroxidation in animal tissues, significantly decreased following supplementation with black currant extracts [81]. In the study by Zhao et al., the distribution of Nrf-2 in hepatocytes of mice fed a high-fat diet supplemented with exosome-like nanoparticles from blueberry was determined. The supplementation accelerated the translocation of Nrf-2 from the cytoplasm to the nuclei in the livers of mice fed a high-fat diet. The expression of Bcl-2 and HO-1 increased in the livers of C57BL/6 mice fed a high-fat diet supplemented with blueberry, while the level of Bax protein decreased [78]. Conversely, the studies by Hewage et al. [32] demonstrated that supplementing with 2% or 5% (*w*/*w*) *Vaccinium vitis-idaea* L. (lingonberry) restored nuclear Nrf-2 protein levels in the liver. Additionally, the study measured the levels of genes and proteins involved in glutathione synthesis, specifically glutamate-cysteine ligase (Gclc—the catalytic subunit and Gclm—the modifier subunit) and glutathione synthetase. Lingonberry supplementation increased the expression of Gclc and the mRNA levels of Gclm in the liver.

Oxidative stress biomarkers encompass malondialdehyde (MDA) and reduced glutathione (GSH). During oxidative stress, intracellular superoxide species are produced, contributing to liver damage. The liver’s antioxidant enzyme, superoxide dismutase (SOD), is essential for removing these harmful species [91,92]. Antioxidant enzymes, including SOD, catalase (CAT), and glutathione peroxidase (GPx), increased in response to supplementation with extracts from plants of the *Vaccinium* genus [32,35,75,76,78,81]. Rats administered cranberry exhibited a marked increase in SOD and catalase activities, as well as depleted GSH levels. A higher dose of cranberry (100 mg/kg) significantly restored depleted GSH by 257% compared to the high-fat diet group, in which SOD and GSH levels had decreased by nearly 80% [71]. Similarly, in the studies by Li et al., blueberry leaf extract inhibited the increase in MDA and reduced ROS levels in the liver [35]. Feeding mice a high-fat diet supplemented with lingonberry reduced MDA levels and restored GSH levels. Lingonberry supplementation also decreased hepatic GSSG levels and restored the GSH/GSSG ratio [32].

Additionally, Shimizu et al. measured the mRNA levels of IL-6, a proinflammatory cytokine, as an indicator of oxidative stress. Their results indicated that cranberry treatment tended to suppress IL-6 expression and reduce oxidative stress in the liver [84].

#### 3.4.5. *Vaccinium*, Lipid Metabolism, and NAFLD

**Figure 3:**
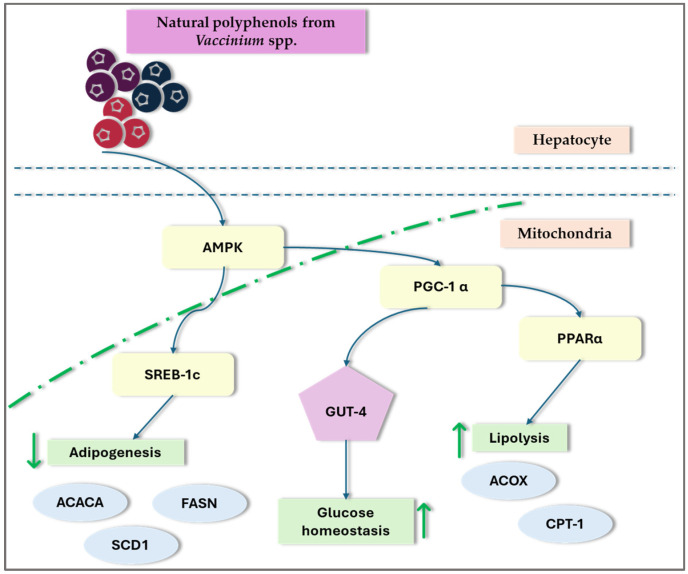
General overview of the impact of *Vaccinium* spp. on transcription factors. ACC—acetyl-coenzyme A carboxylas; ACOX1—acyl-CoA oxidase1; ADP—adenosine-5′-diphosphate; AMPK—AMP-activated protein kinase; CPTIα—carnitine palmitoyltransferase-I-alpha; PPAR—peroxisome proliferator-activated receptor; SREBP—sterol regulatory element-binding protein; ↑—increase; ↓—decrease;.

Recent research on NAFLD and lipid metabolism disorders has indicated that disruptions in hepatic lipid metabolism lead to lipid accumulation, resulting in hepatotoxicity and NAFLD. Dyslipidemia, marked by elevated levels of FFA, ox-LDL, and TG in the plasma, contributes to inflammation, oxidative stress, lipotoxicity, and liver damage. This condition can manifest at any stage of NAFLD and can worsen its progression [3]. Peroxisome proliferator-activated receptors (PPARs) and sterol regulatory element-binding proteins (SREBPs) are crucial for regulating lipid metabolism and are significant in the pathogenesis of NAFLD. Studies indicate that certain *Vaccinium* species, such as blueberries and cranberries, may affect these transcription factors and potentially offer therapeutic benefits in NAFLD by modulating AMPK signaling (Figure 3) [9,31,32,35,71,75,93].

##### Impact of *Vaccinium* on Peroxisome Proliferator-Activated Receptors (PPARs)

Peroxisome proliferator-activated receptors (PPARs) are ligand-activated transcription factors in the nuclear receptor family. They regulate lipid metabolism, glucose homeostasis, energy balance, inflammation, and atherosclerosis. PPARs are divided into three isoforms: alpha (α), beta (β)/delta (δ), and gamma (γ), each with distinct expression patterns across different tissues [94]. The three PPAR isotypes have unique lipid and glucose metabolism roles, crucial in developing NAFLD. PPAR-α is a crucial regulator of fatty acid breakdown in the liver, overseeing the expression of genes involved in the body’s overall balance of fatty acids [95]. Evidence suggests that PPAR-α can regulate the expression of key transcription factors SREBP-1c and liver X receptor α (LXRα), thereby enhancing the transcription levels of Scd-1 and other lipogenic genes, ultimately affecting hepatic lipogenesis [96].

In the liver, PPAR-β/δ regulates lipid and glucose metabolism, with its expression significantly reduced during fasting but rapidly restored upon refeeding. The activation of PPAR-β/δ enhances insulin sensitivity in diabetic mice, mainly through the modulation of genes involved in hepatic fatty acid production and the pentose phosphate pathway [95]. PPAR-β/δ may play a crucial role in regulating lipid metabolism [88,94,97].

PPAR-γ promotes insulin sensitivity by regulating lipid storage in adipose tissue and the differentiation of adipocytes. PPAR-γ is predominantly active during the postprandial state and regulates fat storage in adipose tissue. As a transcription factor, PPAR-γ controls the expression of genes involved in adipogenesis, adipose tissue differentiation, and lipid metabolism, including fatty acid uptake and triacylglycerol lipolysis in adipose tissue. Consequently, tissue-specific deficiency of PPAR-γ leads to significant loss of adipose tissue and severe insulin resistance, resulting in hepatic fat accumulation [88,98].

In an immunohistochemical analysis conducted by Ren et al. [75] to assess the impact of blueberry juice on the expression of PPAR-α in the liver of NAFLD rats, PPAR-α in the NAFLD group exhibited weak expression in the cytoplasm with a very light brown color. In contrast, in the blueberry-juice-supplemented group, PPAR-α showed strong expression in the cytoplasm with a brown color. Regarding the expression of SIRT1, SREBP-1c, and PPAR-α in the control group, the SIRT1 and PPAR-α proteins were high, while the level of SREBP-1c was low. In contrast, in the liver of NAFLD rats, SIRT1 and PPAR-α protein levels were low, while SREBP-1c levels were high. In the blueberry-juice-treated group, the SIRT1 and PPAR-α protein levels were high, whereas the level of SREBP-1c was low. In a separate study, Ren et al. [76] validated the impact of blueberry juice on PPAR-α and subsequently on the SREBP-1c/PNPLA-3 pathway. Their findings indicate that blueberry juice elevated PPAR-α protein levels, whereas blocking PPAR-α activity increased SREBP-1c and PNPLA-3 levels. Thus, blueberry juice may help inhibit the progression of NASH by modulating the SREBP-1c-PNPLA-3 pathway via PPAR-α. The hepatic expression of peroxisome proliferator-activated receptor α was significantly reduced by 24% due to supplementation with *Vaccinium macrocarpon* extract [33]. In the study by Shimizu et al., hepatic mRNA levels of PPAR-γ and MCP-1, which are detected in patients with NAFLD, were significantly lower in mice fed a high-fat diet supplemented with 5% cranberry powder compared to mice on a high-fat diet alone. This implies that supplementing with cranberry powder can mitigate NAFLD caused by a high-fat diet [84]. In studies investigating the impact of *Vaccinium vitis-idaea* L. supplementation on hepatic gene expression in mice fed a high-fat diet, it was confirmed that this diet significantly increased the expression of PPAR-γ and its target genes, such as monoacylglycerol O-acyltransferase 1 (Mogat1), the cluster of differentiation 36 (CD36), and death effector domain-containing similar to DFFA inducing apoptosis c (Cidec). Supplementation with cranberry inhibited the increase in expression of the monoacylglycerol O-acyltransferase 1 (Mogat1) gene, which plays a role in lipid metabolism and is activated by PPAR-γ [80].

##### Impact of *Vaccinium* on Sterol Regulatory Element-Binding Proteins (SREBPs)

Sterol regulatory element-binding proteins are transcription factors involved in cholesterol, TG, and FFA biosynthesis. SREBPs are categorized into three subtypes: SREBP-2, SREBP-1c, and SREBP-1a [99]. These factors are integral to the development of conditions such as non-alcoholic fatty liver disease, non-alcoholic steatohepatitis, and liver cancer. In their phosphorylated state, SREBPs remain inactive in the cytoplasm, but once dephosphorylated, they move to the nucleus, promoting lipogenic enzyme production. Among them, SREBP-1c is a vital regulator of liver lipogenesis [100,101]. PPAR-γ and CCAAT/enhancer-binding proteins alpha (C/EBPα) are other transcription factors involved in the differentiation of adipocytes and lipid accumulation [102,103].

Ren et al. [75] suggest that blueberry juice may improve NAFLD by activating the SIRT1-mediated signaling pathway. Their research demonstrated that blueberry juice can enhance the expression of SIRT1, increase the levels of PPAR-α protein, and reduce the levels of SREBP-1c. The same authors’ studies showed that blueberry juice increased PPAR-α protein levels and inhibited PPAR-α activity, which increased SREBP-1c and PNPLA-3 levels. This indicates that blueberry juice could potentially slow the advancement of NASH by modulating the SREBP-1c/PNPLA-3 pathway via PPAR-α [76]. Peroxisome proliferator-activated receptor, gamma coactivator 1a (PGC-1a), and sirtuin-3 (SIRT3) are regulators of AMP-activated protein kinase (AMPK), increasing their expression through AMPK-mediated phosphorylation. PGC-1a further enhances the activity of various nuclear receptors, such as estrogen-related receptor alpha (ERRα), which is crucial for mitochondrial biogenesis and Nrf-1 and Nrf-2. Additionally, SIRT3 is involved in the activation of antioxidant enzymes. In a particular study, administering 100 or 400 mg/kg body weight per day of a 70% ethanol extract from *Vaccinium corymbosum* L. leaves by oral gavage activated the AMPK/PGC-1a/SIRT3 signaling pathway in rats that were on a high-fat diet for 9 weeks [35]. A high-fat diet induces lipid accumulation in the liver, leading to increased expression of SREBP-1c and ACC-1. Lingonberry supplementation attenuated the high-fat-diet-induced expression of SREBP-1c and ACC-1, reduced plasma lipid levels, and improved hepatic steatosis. The study findings imply that the decrease in hepatic lipid accumulation observed with lingonberry supplementation could be attributed to the suppression of SREBP-1c [32]. In the study by Hewage et al. [31], lingonberry supplementation was found to inhibit the expression of SREBP-1c and ACC1 in the livers of mice on a high-fat diet, leading to a reduction in lipogenesis. The study also noted a decrease in the expression of the enzymes DGAT1 and DGAT2, which may contribute to lower triglyceride levels in the livers of these mice. Furthermore, lingonberry supplementation reduced the expression of CD36, a receptor crucial for fatty acid uptake, which could further explain the decrease in hepatic fatty acid absorption.

##### Influence of *Vaccinium* on AMP-Activated Protein Kinase (AMPK)

AMP-activated protein kinase is a key sensor and regulator of cellular energy balance. It plays an essential role in managing lipid and glucose metabolism, as well as controlling oxidative stress and inflammatory responses within the body [104]. Previous research has shown that the inhibition of AMPK signaling is associated with the progression of NAFLD [105]. AMPK plays a crucial role in regulating lipid metabolism by influencing various processes such as the oxidative breakdown of FFA and TG and their synthesis. This regulation supports cellular metabolism and proliferation, making AMPK a key player in the development of fatty liver disease [106]. AMPK reduces hepatic fat accumulation through two mechanisms. The first mechanism involves the phosphorylation and inactivation of acetyl-CoA carboxylase (ACC) by activated AMPK, preventing ACC dimerization and reducing fatty acid synthesis. The second mechanism is related to malonyl-CoA, which serves as a precursor for fatty acid synthesis and a potent carnitine palmitoyltransferase 1 (CPT1) inhibitor. AMPK phosphorylates ACC, decreasing malonyl-CoA levels, which promotes CPT1 expression and thus enhances fatty acid oxidation [106,107].

Extracts from *Vaccinium* species may influence the activity of fatty acid synthase (FAS) and ACC. In vivo, studies demonstrated that a high-fat diet significantly reduced pAMPKα, PGC-1α, and SIRT3 proteins in rat livers, an effect that was effectively countered by the administration of blueberry leaf extracts [35]. In a study on de novo hepatic lipogenesis, the phosphorylation of AMPK was evaluated by measuring the ratio of phosphorylated AMPK (pAMPK) to total AMPK protein levels in the liver. A high-fat diet significantly decreased both pAMPK levels and the pAMPK/AMPK ratio compared to animals on a control diet. However, supplementation with lingonberry restored both pAMPK levels and the pAMPK/AMPK ratio [32].

#### 3.4.6. The Effect of *Vaccinium* on Glucose Metabolism

Abnormal glucose metabolism and insulin resistance (IR) are also recognized as key risk factors in the development and progression of NAFLD [108]. Insulin resistance is marked by a diminished capacity of tissues outside the liver, such as adipose tissue and muscles, to use glucose effectively. In adipose tissue, this resistance results in abnormal fatty acid release due to disrupted lipolysis, exacerbating insulin signaling impairment across the entire body [3].

In research conducted by Glisan et al. [33], cranberry supplementation did not significantly affect fasting plasma insulin levels, fasting blood glucose levels, or HOMA-IR. Similarly, in research by Shimizu et al. [84], fasting blood glucose levels were higher in mice fed a high-fat diet, and glucose levels did not differ significantly between the groups receiving 1% and 5% doses of cranberry. In contrast, in studies by Haga et al. [74], plasma glucose and TC levels in the group receiving a 10% extract of *Vaccinium myrtillus* L. were significantly lower than those in the high-fat and high-cholesterol diet group. Nakano et al. [81] observed a significant reduction in serum insulin levels and insulin resistance with supplementation of *Vaccinium myrtillus* L. extracts; however, glucose levels did not differ significantly across all studied groups. HOMA-IR and TyG index, FGIR index values, and pancreatic β-cell function did not show statistically significant differences for the high-fat and high-fructose diets. Conversely, studies by Faheem et al. [36] demonstrated a significant reduction in the HOMA-IR index in rats treated with cranberry at doses of 50 and 100 mg/kg, showing decreases of 54% and 77%, respectively. Additionally, the TyG index was notably lowered by 6% and 11% in the same cranberry-treated groups. Blueberry-derived exosome-like nanoparticles (BELNs) improved insulin resistance, reduced fasting glucose levels, and decreased serum insulin content in mice fed a high-fat diet. The results demonstrated that C57BL/6 mice could take up BELNs and they were rapidly distributed into several organs, including the small intestine, liver, and spleen. These findings suggest that the administration of BELNs may be beneficial in ameliorating the pathological features of NAFLD [78].

**Table 2:** Studies evaluating the influence of *Vaccinium* spp. on non-alcoholic fatty liver disease in vivo.

Study	Study Types		Type of Intervention	Effects	STAIR
	Animal Model	Dosage and Duration		Metabolism/Molecular	
Ren, Huang, and Cheng 2014 [75]	Male Sprague Dawley rats (200 to 250 g)	Blueberry juice (15 g/kg, once a day) / 8 weeks	Rats were divided into 2 groups: (1) HFD—50 rats; (2) control group—8 rats	↓ The degrees of NAFLD and degenerated hepatocytes; ↓ Serum activities of AST and ALT; ↓The ratio of TG/HDL-c; ↑ The mRNA levels of SIRT1, PPAR-α; ↓ The levels of SREBP-1c.	5
Morrison et al. 2015 [72]	Female ApoE 3Leiden mice	0.1% (*w*/*w*) Mirtoselect—standardized *Vaccinium myrtillus* L. extract (36% anthocyanins)/20 weeks	Mice were divided into 3 treatment groups: (1) HCD; (2) HCD and 0.1% (*w*/*w*) Mirtoselect; and (3) Western-type diet without cholesterol supplementation	↓ The development of hepatic steatosis; ↓ Microvesicular steatosis; ↓ An accumulation of lipids esterified to cholesterol (cholesteryl esters); ↓ The hepatic free cholesterol; ↓ p65-NF-κB activity; Expression of *Emr1* or *Ccl2*—not significant; ↓ Neutrophil infiltration and the expression of two neutrophil chemoattractants—*Cxcl1* and *Cxcl2*; ↓ The pronounced increase in collagen and significantly reduced *Col1a1* expression.	4
Glisan et al. 2016 [33]	Male C57BL/6J mice (4 weeks old)	0.8% CBE—4 g per day (CBE, D13051702) (CBE—macerated sulfite-free dried cranberries)/21 weeks	Mice were divided into 2 treatment groups: (1) HFD (n = 24) and (2) CBE diet (n = 24) for 10 weeks	Blood glucose levels, plasma insulin levels, and HOMA-IR—not significant; ↓ The plasma levels of free fatty acids; ↓ The plasma levels of IL-1β; ↓ The serum levels of ALT levels; ↓ The total lipid droplet area in the liver and the total hepatic lipid area; ↓ The hepatic expression of the NF-κB-dependent proinflammatory genes TNF-α (↓47%) and Cox2 (↓46%); ↓ The hepatic mRNA expression of Il1b (55%) and Ucp2 (57%); ↓ The hepatic mRNA levels of C-C chemokine receptor 2 (Ccr2), the CCL2 receptor expressed on recruited monocytes, by 56% and Ccl3 by 55%; ↓ The hepatic expression of Nlrp3 (43%) and Txnip (30%); ↑ The gene expression of hepatic PPAR-α. ↓ The transcription factor responsible for regulating Txnip expression (24%).	5
Ren et al. 2017 [76]	Male Sprague Dawley rats (6 to 8 weeks old)	10 mL/kg blueberry juice (1 kg blueberries were thawed, milled, and pressed)/12 weeks	Rats were divided into 2 groups: (1) blueberry juice group (injected with 50 μL/kg saline solution and orally received 10 mL/kg blueberry juice and 10 mL/kg liquid placebo daily); (2) blueberry juice and PPAR-α inhibitor group (injected with 50 μL/kg PPAR-α in saline solution and orally received 10 mL/kg blueberry juice and 10 mL/kg liquid placebo daily)	Blueberry juice: ↓ The serum levels of ALT and AST; ↑ The levels of SOD and GSH; ↓ The serum levels of MDA, TG, TC, and LDL-C increased HDL-C levels; ↑ the mRNA levels of PPAR-α, which reduced the level of SREBP-1c and PNPLA-3. Blueberry juice and PPAR-α inhibitor: ↓ the mRNA levels of SREBP-1c and PNPLA3-α.	5
Shimizu et al. 2019 [84]	Male mice C57BL/6 (6 weeks old)	1% cranberry powder or 5% cranberry powder (anthocyanin 120 mg/100g and proanthocyanidin 2600 mg/100 g)/8 weeks	Mice were divided into 4 treatment groups: (1) ND, (2) HFD, (3) HFD + 1% cranberry powder, and (4) HFD + 5% cranberry powder	↓ Body weight and concomitantly triggered hyperphagia; ↓ Oxidative stress and proinflammatory cytokine expression (IL-6); The serum levels of glucose—not significant; ↓ The serum levels of TG; ↓ The serum level of ALT; ↓ The serum level of hepatic mRNA of PPAR-γ and MCP-1.	4
Haga et al. 2019 [74]	Male homozygous leptin receptor-deficient (BKS.Cg-+ Leprdb/+ Leprdb/Jcl; db/db) mice (10 weeks old)	5% and 10% bilberry fruits extracts (≥36% anthocyanin glycosides)/8 weeks	Mice were divided into 4 treatment groups: (1) ND, (2) HFD + HCD, (3) HFD + HCD + 5% bilberry fruit extracts, and (4) HFD + HCD + 10% bilberry fruit extracts	↓ Fat accumulation and TG contents in mouse liver; Less fibrosis; ↓ The serum levels of ALT and AST; ↓ The plasma levels of GLU and TC; ↓ Proinflammatory cytokine levels (TNF-α, IL-9, IL-1β, and IFN-γ).	4
Li et al. 2020 [35]	Male Sprague Dawley rats (8 weeks old)	Freeze-dried leaf extract of *Vaccinium corymbosum* L. (PBL)/8 weeks	Mice were divided into 4 groups: (1) ND; (2) HFD (3) HFD + high dose PBL (H-PBL); (4) HFD + low dose PBL (L-PBL). Rats received: PBL at a dose of 400 mg kg/day (H-PBL group) or 100 mg/kg/day (L-PBL group) or an equal volume of vehicle (0.9% NaCl, ND, and HFD groups) by gavage for 9 weeks	↓ The hepatic TC, TC, L-LDL, ALT, and AST levels; ↓ The hepatic steatosis and inflammatory infiltration; ↓ The generation of hepatic malondialdehyde (MDA); ↓ The hepatic ROS levels; Protection against hepatic oxidative stress; ↓ pAMPKα, PGC-1α, and SIRT3 proteins in liver; ↑ ERRα, Nrf-1, and Nrf-2 genes in liver.	5
Nakano et al. 2020 [81]	Male mice C57BL/6N (5 weeks old)	2% bilberry anthocyanin extract powder (Mirtoselect)/18 weeks	Mice were randomly divided into 4 groups: (1) ND group, (2) ND + 2% bilberry anthocyanins, (3) WD group, and (4) WD + 2% bilberry anthocyanins	↓ Body weight, liver weight, epididymal fat mass, liver-to-body-weight ratio, and hepatic fat mass; ↓ The serum level of AST and ALT; ↓ The serum level of MCP-1; ↓ The serum level of TC; ↓ The serum level of insulin; ↑ Insulin resistance; The serum levels of HDL-c, TG, and glucose—not significantly different; ↑ The level of lactic acid in the gut; ↑ The levels of Nrf-2 and SOD2; ↓ The level of Keap1 and TBARS in the liver.	5
Faheem et al. 2020 [71]	Male albino Wistar rats (12 weeks old)	Cranberry nutraceutical (186c1025) diluted in water (40 mg/mL)/8 weeks	Mice were divided into 5 groups: control group: (1) ND for 8 weeks and received 1 mL/kg distilled water orally thrice weekly; HFCD group: (2) HFCD for 8 weeks and received 1 mL/kg distilled water orally three times weekly; 50/HFCD group: (3) HFCD and cranberry (50 mg/kg/day) orally three times weekly; 100/HFCD group: (4) HFCD and cranberry (100 mg/kg/day) orally three times weekly; treated group: (5) ND and cranberry (100 mg/kg/day) orally three times weekly	↓ Body weight; ↓ The serum levels of ALT and AST; ↓ The serum levels of TG; ↓ HOMA IR; ↑ SOD and GSH; ↑ ADP levels; ↑ Nrf-2; ↓ The serum level of TNF-α, IL-6, NF-κB, ↓ TGF-β and α-SMA tissue levels; ↓ Reduction of collagen deposition; ↑ IRS-2 expression.	5
Zhao et al. 2021 [78]	Male mice C57BL/6 (6–8 weeks old)	Blueberry-derived exosome-like nanoparticles (BELNs) at 25, 50, or 100 mg/kg/4 weeks	Mice in the 3 HFD groups received intragastric administration of blueberry-derived exosome-like nanoparticles at doses of 25, 50, or 100 mg/kg, administered once every other day	↓ The serum level of insulin, fasting glucose; ↑ Insulin resistance; ↓ The accumulation of lipid droplets in the liver and the liver weight; ↓ The contents of TC and TG, the levels of ALT and AST, and LDL-C; ↑ The content of HDL-C; ↑ The activities of SOD and GSH; ↓ The content of MDA in the liver; Accelerated the translocation of Nrf-2 from the cytoplasm to nuclei in the liver; ↓ The mRNA levels of FAS and ACC1 in the liver; ↓ The expression of Bcl-2, Bax, and HO-1 in the liver.	5
Hewage et al. 2021 [32]	Male C57BL/6J mice (6 weeks old)	(5% *w*/*w*) Manitoba lingonberry *Vaccinium vitis-idaea* L./freeze-dried berry powder/12 weeks	Mice were divided into 3 groups: (1) control (D12450J) diet, (2) HFD (D12492), or (3) HFD supplemented with (5% *w*/*w*) Manitoba lingonberry	↓ The serum levels of ALT and AST; ↓ The hepatic accumulation of TG and TC; ↓ MDA levels and restored GSH levels; ↓ The hepatic GSSG level and restored GSH/GSSG ratio; ↓ The hepatic ACC-1, SREBP-1c mRNA expression, and the nuclear protein level of SREBP-1c; ↑ The expression of Gclc in the liver; ↓ The serum levels of IL-6, MCP-1, and TNF-α mRNA expression; ↑ pAMPK level and pAMPK/AMPK ratio; ↑ Nuclear Nrf-2 protein level in the liver.	4
Ryyti et al. 2021 [80]	Male C57BL/6N mice (8 weeks old)	20% *w*/*w* air-dried lingonberry *Vaccinium vitis-idaea* L. powder (900 g of fresh lingonberries were used to produce 100 g of berry powder)/6 weeks	Mice were divided into 3 groups: (1) LFD (10 kcal% fat); (2) HFD (46 kcal% fat); (3) HFD with air-dried lingonberry powder (20% *w*/*w*)	↓ The serum levels of ALT; ↓ The expression of the acute phase inflammatory factors Saa1 and Saa2; ↓ The expression of Cyp46a1; ↑ The expression of hydroxysteroid (17-beta) dehydrogenase 6 (Hsd17b6) and insulin-like growth factor binding protein 2 (Igfbp2); ↓ The expression of genes associated with lipid metabolic process (Mogat1, Plin4), inflammatory/immune response or cell migration (Lcn2, Saa1, Saa2, Cxcl14, Gcp1, S100a10), and cell cycle regulation (Cdkn1a, Tubb2a, Tubb6).	4
Hewage et al. 2022 [31]	Male C57BL/6J mice (6 weeks old)	5% w/w Manitoba wild lingonberry/12 weeks	Mice were divided into 3 groups: (1) a control diet (D12450J) containing 11% kcal fat, 18% kcal protein, and 71% kcal carbohydrate, or (2) an HFD (D12492) containing 62% kcal fat, 18% kcal protein, and 20% kcal carbohydrate, or (3) an HFD supplemented with (5% *w*/*w*) Manitoba wild lingonberry	↓ The hepatic accumulation of TG and TC; ↓ Notch1 expression in the liver; ↓ Liver NICD1 protein and HES1 mRNA levels; ↓ The expression of SREBP-1c and ACC1; ↑ The hepatic mRNA levels of ACOX1 and CPTIα; ↓ Gene expressions of CD36, DGAT1 and DGAT2.	3
Zhu et al. 2022 [77]	Male C57BL/6 mice (8–10 weeks old)	TEC–blueberry monomers were prepared as 1000 ppb, 800, 600, 400, 200, and 100 ppb with 0.1% formic acid methanol solution/16 weeks	Mice were treated with TEC via gavage at doses of 7.5, 15.0, or 30.0 mg/kg daily for 6 weeks following 10 weeks on a high-fat diet (HFD). To achieve tRF-47 knockdown in vivo, a tRF-47 antagomir (5 μg/mouse in 1.5 mL saline) was injected into the tail vein of NASH mice three times a week for 2 weeks, starting after 9 weeks on the HFD	↓ The serum levels of ALT, AST and MDA; ↑ The level of autophagy marker LC3B in the liver; ↓ The activation of inflammasomes and TLR4; TEC relies on tRF-47 (tRF-47-58ZZJQJYSWRYVMMV5BO) to promote autophagy and weaken pyroptosis.	5
Sotelo-González et al. 2023 [82]	Male Wistar rats	10% (w/v) blueberry aqueous extracts/18 weeks	Mice were divided into 3 groups: (1) standard-diet-fed group; (2) HFFD (standard diet added with 20% lard and 18% fructose); (3) HFFD with blueberry beverage	↓ The serum level of TG; ↓ The accumulation of lipid vacuoles; ↓ The accumulation of saturated, monounsaturated, and polyunsaturated fatty acids; ↓ FAS and ACC expression.	5

ACC—acetyl-coenzyme A carboxylas; ACOX1—acyl-CoA oxidase1; ADP—adenosine-5′-diphosphate; ALT—alanine aminotransferase; AMPK—AMP-activated protein kinase; AST—aspartate transaminase; CD36—fatty acid translocase; CPTIα—carnitine palmitoyltransferase-I-alpha; DGAT (1 and 2)—diacylglycerol O-acyltransferase; FAS—fatty acid synthase; GSH—glutathione; GPx—glutathione peroxidase; HCD—high-cholesterol diet; HDL—high-density lipoprotein; HES1—hairy and enhancer of split-1; HFD—high-fat diet; HFCD—high-fat and -cholesterol diet; HFFD—high-fat and -fructose diet; HOMA-IR—homeostatic model assessment for insulin resistance; IL—interleukin; IFN-γ—interferon gamma; IRS-2—insulin receptor substrate; Keap1—Kelch-like ECH-associated protein 1; LDL—low-density lipoprotein; MCP-1/CCL2—monocyte chemoattractant protein-1; MDA—malondialdehyde; ND—normal diet; NOS—nitric oxide synthase; NF-κB—nuclear factor kappa B; NLRP3—NLR family pyrin domain containing 3; Notch 1—neurogenic locus notch homolog protein 1; Nrf-1—nuclear respiratory factor 1; Nrf-2—nuclear factor erythroid 2-related factor 2; PNPLA—patatin like phospholipase domain containing; PPAR—peroxisome proliferator-activated receptor; SAA—circulating serum amyloid A; SOD—superoxide dismutase; SREBP—sterol regulatory element-binding protein; STAT3—signal transducer and activator of transcription; TC—total cholesterol; TG—triacylglycerol; TGF-β—transforming growth factor β; Txnip—thioredoxin-interacting protein; TLR4—Toll-like receptor 4; TNF-α—tumor necrosis factor alpha; WD—Western diet; ↑—increase; ↓—decrease.

### 3.5. Cell Culture Experiments

Studies evaluating the effects of *Vaccinium* species in vitro on lipid metabolism and oxidative stress in hepatocytes, typical of NAFLD and NASH, are presented in Table 3. Most studies were conducted on human hepatoma HepG2 cells [31,35,74,77,79,109,110]. In all studies, extracts rich in polyphenols from *Vaccinium* berries were utilized. Unfortunately, the interpretation of results is complicated by variations in the cell models, experimental protocols, and molecular pathways assessed. Despite these differences, most studies agree that extracts from blueberries or cranberries help reduce hepatocyte lipid accumulation by inhibiting lipogenesis and likely enhancing lipolysis. However, not all studies have explored all facets of lipid metabolism. Despite the variability in cell models and experimental methods, most research consistently supports the beneficial effects of these extracts on lipid reduction and the enhancement of mitochondrial function in hepatocytes.

Liu et al. [79] investigated the inhibitory effect of blueberry polyphenol-rich extract and its fractions on TG accumulation in HepG2 cells. They found that at a concentration of 80 μg/mL, the extract achieved a maximum inhibition of TG synthesis of approximately 60%. To identify the most effective component in reducing lipid accumulation, the extract was divided into three fractions: an anthocyanin-rich fraction, a phenolic-acid-rich fraction, and an ethyl acetate extract. The phenolic-acid-rich fraction demonstrated the greatest efficacy in TG clearance, reducing levels by 58.6 ± 4.7% at a 100 μg/mL concentration. The anthocyanin-rich fraction reduced TG levels by 30% at the same concentration. In contrast, the ethyl acetate extract showed the least bioactivity in inhibiting TG accumulation in HepG2 cells. Wang et al. [109] also assessed the inhibitory effects of wild blueberry extracts on TG accumulation and ROS levels in HepG2 cells. In their study, HepG2 cells were treated with blueberry extract at concentrations of 10, 20, and 40 μg/mL alongside oleic acid for 24 h to assess the impact on lipid accumulation. The blueberry extracts led to a 24% reduction in TG levels and decreased ROS levels in liver cells. Additionally, bilberry extract enhanced the growth of steatotic hepatocytes induced by oleic and linoleic acids or the nuclear receptor LXRα agonist, with optimal effects at a concentration of 5 μg/mL. The extracts reduced lipid deposition in steatotic hepatocytes, promoted cell growth, protected against oxidative stress, and significantly inhibited lipid accumulation. Moreover, when AML12 cells were exposed to fatty acids, Rubicon expression was markedly increased. The addition of bilberry extracts notably reduced Rubicon expression and significantly increased p62/SQSTM expression, indicating enhanced autophagy and a subsequent reduction in lipid accumulation [74]. Blueberry leaf extracts effectively alleviated the reduction of mitochondrial membrane potential in the mitochondria of fatty-acid-induced cells. Additionally, the extracts improved mitochondrial OCR, ATP production, and basal and maximal respiration in HepG2 cells. Blueberry extracts support the mitochondrial energy supply in the liver by increasing the pools of mitochondrial NTP and NMP in HepG2 cells. The balance of mitochondrial deoxyribonucleotide triphosphate (dNTP) pools is critical for the fidelity of DNA synthesis during mtDNA replication and repair. The extracts significantly mitigated the reduction of mtDNA in both cells and the livers of rats. In the same study, blueberry leaf extracts significantly increased the phosphorylation of AMPKα, the expression of SIRT3 protein, the expression of PGC-1α protein, and the expression of ERRα, Nrf-1, and Nrf-2 genes in fatty-acid-induced HepG2 cells [35]. In the study by Wang et al. [110], HepG2 cells treated with blueberry leaf extract at concentrations ranging from 0.78 to 25 µg/mL showed increased viability compared to NAFLD-modeled cells. Additionally, the extracts reduced lipid accumulation in the cells. The results indicated that PBL treatment might suppress NAFLD-induced apoptosis, as it decreased the number of apoptotic cells. Specifically, the leaf extract at a concentration of 25 µg/mL significantly reduced the expression of the caspase-3 protein and slightly increased the expression of the antiapoptotic protein Bcl-2. Blueberry-derived exosome-like nanoparticles (BELNs) notably increased mitochondrial content and reduced ROS production in a dose-dependent manner in HepG2 cells treated with rotenone. Under rotenone treatment, these cells displayed a decreased mitochondrial membrane potential (MMP), but preincubation with BELNs effectively mitigated this effect. Mitochondria, key organelles for energy production via oxidative phosphorylation, depend on MMP as a crucial driving force. Dose-dependent preincubation with BELNs also counteracted the effects of rotenone on the protein levels of Bcl-2, Bax, and HO-1 in HepG2 cells, significantly decreasing rotenone-induced apoptosis. This suggests that Nrf-2 is an essential transcription factor that activates the expression of various antioxidant enzymes and proteins by relocating from the cytoplasm to the nucleus in mammalian cells exposed to oxidative stress [78]. Hewage et al. [31] investigated the impact of lingonberry on Notch1 signaling and lipid metabolism by exposing hepatocytes to palmitic acid. They observed that palmitic acid induced the expression of the Notch1 gene. Subsequently, they examined the effects of lingonberry extract and C3Glu (cyanidin-3-O-glucoside) on Notch signaling and the induction of genes associated with lipid synthesis. Treatment with lingonberry extract or C3Glu significantly reduced palmitic-acid-induced Notch1 mRNA expression. Furthermore, lingonberry extract or C3Glu lowered the mRNA expression levels of HES1, SREBP-1c, and ACC1 and reduced palmitic-acid-induced intracellular triacylglycerol accumulation. Moderate concentrations of anthocyanin monomers (TEC) enhanced cell viability in the NASH model, decreased the secretion of IL-6, TNF-α, IL-10, IL-17, and IL-4, and normalized the elevated expression of TLR4 associated with the NASH model. Additionally, TEC mitigated pyroptosis by stimulating autophagy, which reduced NLRP3 and GSDMD expression and inhibited the fatty-acid-induced release of LDH [77].

**Table 3:** Studies evaluating the influence of *Vaccinium* spp. on non-alcoholic fatty liver disease in vitro.

Study	Study Types		Effects
	Model	Dosage and Duration	
Liu et al. 2011 [79]	Human hepatocellular cancer cell line (HepG2)	Anthocyanin-rich and phenolic-acid-rich fractions from fresh blueberries (*Vaccinium* spp.)	– ↓ TG deposition in HepG2 cells; – achieved a maximum TG synthesis inhibition rate of approximately 60% (80 μg/mL); – the phenolic-acid-rich fraction was the most effective for TG clearance, with a maximum of 58.6 ± 4.7% at 100 μg/mL; – the anthocyanin-rich fraction achieved a maximum TG clearance of 30% at 100 μg/mL.
Wang et al. 2016 [109]	Human hepatocellular cancer cell line (HepG2)	purified ACNs from wild blueberries (*Vaccinium* spp.); −10, 20, and 40 µg/mL	– ↓ increase in TG levels; – ↓ the rise in ROS in hepatic cells; – ↓ the number of lipid droplets in cells treated with Dp-3-Glu; – ↓ oleic acid-induced lipid accumulation in hepatic cells.
Haga et al. 2019 [74]	Alpha mouse liver 12 cells	A 90% ethanolic extract of bilberry fruits (≥36% anthocyanin glycosides)	– ↓ cellular lipid accumulation in both types of hepatic steatosis (FA and T090); – ↑ cell survival/proliferation with a peak effect at 1 μg/mL; – ↓ Rubicon (protein inhibiting late-stage autophagy) – ↑ p62/SQSTM1 (autophagy marker); – ↓ the cellular FA synthesis pathway stimulated by the LXR agonist (nuclear receptor transcription factor involved in lipid synthesis);
Li et al. 2020 [35]	Human hepatocellular cancer cell line (HepG2)	Blueberry (*Vaccinium corymbosum* L.) leaves (PBL);3 doses of PBL at 10 (high), 5 (medium), and 2.5 (low) μg/mL	– ↓ FFA induced lipid accumulation and cellular injury in HepG2 cells; – ↓ FFA induced increases in TNF-α, IL-1β, and IL-6 mRNA levels in HepG2 cells; – ↓ mitochondrial membrane potential (MMP) observed in FFA-treated cells; – ↑ in mitochondrial NTP and NMP pools following FFA treatment, suggesting an improvement in hepatic mitochondrial energy supply; – ↑ AMPKα phosphorylation in HepG2 cells in a dose-dependent manner, leading to increased PGC-1α expression; – ↑ PGC-1α protein expression in a dose-dependent manner; – ↑ the expression of ERRα, Nrf-1, and Nrf-2 genes in FFA-induced cells.
Zhao et al. 2021 [78]	Human hepatocellular cancer cell line (HepG2)	Blueberry-derived exosome-like nanoparticles (BELNs)—100 µg/mL	– ↑ mitochondrial content and inhibited ROS production dose-dependently in HepG2 cells; – ↓ MMP in HepG2 cells; – ↓ rotenone-induced apoptosis in HepG2 cells; – ↑ the translocation of Nrf-2 from the cytoplasm to the nucleus in HepG2 cells.
Wang et al. 2021 [110]	Human hepatocellular cancer cell line (HepG2)	Blueberry leaves (PBL)—6.25, 12.5, 25 µg/mL for 48 h	– ↓ apoptosis population in a dose-dependent manner compared to NAFLD-modeled cells; – ↓ caspase-3 protein expression and slightly upregulated antiapoptotic protein Bcl-2.
Zhu et al. 2022 [77]	Human hepatoma cell line HepG2	25, 50, and 75 μM C3Glu, myricetin, myricetin 3-o-galactoside, delphinidin, and blueberry monomers (TEC)	– ↓ lipid droplet formation (TEC being more effective than C3Glu); – ↓ the release of IL-6, TNF-α, IL-10, IL-17, and IL-4; – ↑ TLR4 expression in the NASH model compared to the control group, but TEC effectively reversed this increase; – ↓ inflammatory mediators, TLR4, and LDH release levels.
Hewage et al. 2022 [31]	Human hepatoma cells (HepG2, cell line: HB-8065)	5% *w*/*w* Manitoba wild lingonberry	– ↓ palmitic-acid-induced HES1, SREBP-1c, and ACC1 mRNA expression; – ↓ palmitic-acid-induced intracellular TG accumulation; – ↓ cellular lipid droplets compared to cells treated with palmitic acid alone.

ACC—acetyl-coenzyme A carboxylase; AMPK—AMP-activated protein kinase; BCL2—B-cell CLL/lymphoma 2; ERRα—estrogen-related receptor alpha; FFA—fatty acid synthase; C3Glu—cyanidin 3-glucoside; Dp-3-glu—delphinidin-3-glucoside; HDL—high-density lipoprotein; HES1—hairy and enhancer of split-1; IL—interleukin; LDL—low-density lipoprotein; NF-κB—nuclear factor kappa B; NLRP3—NLR family pyrin domain containing 3; NMP—nucleoside monophosphate; Nrf-1—nuclear respiratory factor 1; Nrf -2—nuclear factor erythroid 2-related factor 2; NTP—nucleotide triphosphate; PGC-1α—proliferator-activated receptor γ coactivator 1α; SREBP—sterol regulatory element-binding protein; TC—total cholesterol; TG—triacylglycerol; TGF-β—transforming growth factor β; TLR4—Toll-like receptor 4; TNF-α—tumor necrosis factor alpha; ↑—increase; ↓—decrease.

## 4. Discussion

NAFLD is the most prevalent chronic liver disease globally [111]. The onset and advancement of NAFLD are driven by various interconnected factors that work together in individuals with a genetic predisposition. This condition is linked not only to poor lifestyle and dietary choices but also to metabolic disorders, genetic factors, oxidative stress, and disturbances in the gut–liver axis, all of which can impact the disease’s development and progression. Recent studies describe these mechanisms as part of the “multiple-hit hypothesis” in understanding NAFLD pathogenesis and progression [112]. Insulin resistance is one of the critical factors in the development of NAFLD/NASH, leading to increased hepatic de novo lipogenesis and impaired inhibition of adipose tissue lipolysis, consequently resulting in an increased influx of fatty acids to the liver [113]. Fat accumulates in the liver as TG, which occurs simultaneously with increased lipotoxicity due to high levels of free fatty acids, free cholesterol, and other lipid metabolites [114,115]. Free fatty acids are central to the development of fatty liver disease. They mainly derive from the breakdown of TG in adipose tissue and are transported to the liver through the bloodstream. Another critical source of FFA is de novo lipogenesis, where hepatocytes convert excess carbohydrates, especially fructose, into fatty acids. These fatty acids are metabolized in hepatocytes through mitochondrial beta-oxidation or re-esterified into TG. The TG stored in lipid droplets can undergo lipolysis, releasing fatty acids back into the hepatocyte’s free fatty acid pool. However, when the capacity for beta-oxidation or TG synthesis is exceeded, FFAs can generate lipotoxic compounds, leading to endoplasmic reticulum stress, oxidative stress, and inflammasome activation. These processes contribute to hepatocyte injury, inflammation, and the progressive buildup of extracellular matrix [3,5].

In the case of NAFLD, there is no established pharmacological therapy, and the primary treatment approach involves lifestyle-related therapies, including dietary modifications and physical activity, which are gaining increasing clinical significance. Efforts are underway to discover new treatment options for this disease. This article reviews preclinical and clinical studies evaluating the efficacy of extracts isolated from fruits or leaves of plants from the *Vaccinium* genus in treating NAFLD. Both clinical trials received a score of 5 points, indicating that they meet high methodological standards in terms of randomization, blinding, and reporting of withdrawals. In this context, there is no clear distinction in quality between these studies based on the Jadad scale. Preclinical studies have received ratings ranging from 4 to 5 according to the STAIR guidelines. Results from studies rated 4 suggest that these studies are well-conducted but may have minor limitations that do not significantly affect the overall reliability of the results. Such studies are considered solid and may be a good indicator of the potential efficacy of the therapy, but additional validation is recommended before advancing to clinical trials. Studies rated 5 are regarded as high quality, and further research should focus on confirming the results across different models or conditions and potentially initiating the planning of clinical trials.

The analysis of the collected results suggests that berry leaf and fruit extracts may prevent liver steatosis and its progression to NASH. Additionally, compounds in these extracts, such as anthocyanins, may act through various mechanisms to improve NAFLD. Hepatocytes, which contain many mitochondria, are susceptible to dysfunction from excessive production of ROS. This ROS overproduction can cause lipid peroxidation and damage mitochondrial DNA and membranes [89]. Analysis of the results showed that extracts from blueberry, bilberry, and lingonberry, exosome-like nanoparticles derived from blueberry, and blueberry monomers reduced ROS production, increased mitochondrial content and membrane potential, and prevented cell apoptosis by regulating the expression of Bcl-2, Bax, and HO-1 in HepG2 cells [31,35,74,78,109]. Furthermore, extracts from *Vaccinium* plants effectively reduced MDA content, increased SOD and GSH activity, enhanced Bcl-2 and HO-1 protein expression, and decreased Bax protein levels in the livers of mice fed a high-fat diet [9,32,35,71,78,81]. The analysis of the collected studies suggests that *Vaccinium* fruits may be beneficial in treating NAFLD due to their antioxidant and antiapoptotic effects.

Nrf-2 is a crucial transcription factor that orchestrates the adaptive antioxidant response and the cellular reaction to xenobiotic stress. It is pivotal in managing ROS generated during various stress conditions. By regulating the expression of several antioxidant enzymes, including HO-1, SOD, GPx, and CAT, Nrf-2 is thought to contribute to the mitigation of NAFLD [116]. This review demonstrated that blueberry leaf extracts increase Nrf-2 expression, while BELNs enhance Nrf-2 translocation from the cytoplasm to the nucleus in HepG2 cells [35,78]. Studies indicate the beneficial effects of supplementation with various berry extracts on the levels of crucial antioxidant factors and liver enzymes in animals fed a high-fat diet. Supplementation with *Vaccinium corymbosum* L. leaf extract significantly increased the levels of ERRα, Nrf-1, and Nrf-2 genes in the liver. The nuclear level of Nrf-2 increased after supplementation with berry extracts and berry-derived exosome nanoparticles [71,78,81]. These findings suggest that the antioxidant activity of *Vaccinium* may modulate Nrf-2 distribution to induce the expression of antioxidant proteins and enzymes. Li et al. [35] suggest that treating NAFLD with blueberry leaf extract may involve directly neutralizing ROS and activating the AMPK/PGC-1α/SIRT3 signaling pathway, which enhances mitochondrial function and antioxidant defenses. Their research found that blueberry leaf extract significantly increased hepatic pAMPK, PGC-1α, and SIRT3 protein levels and upregulated ERRα, Nrf-1, and Nrf-2 gene expression. This gene activation promotes mitochondrial DNA replication, β-oxidation, antioxidant defense, and oxidative phosphorylation. These combined effects help reduce lipotoxicity, mitigate oxidative damage, and lower inflammation, thereby alleviating NAFLD.

In the pathophysiology of NAFLD, elevated release of proinflammatory cytokines is a frequent occurrence and plays a crucial role in advancing from liver steatosis to more severe stages of the disease [3]. Preclinical studies have shown that extracts from *Vaccinium* fruits and leaves, in various doses, reduce the levels of proinflammatory cytokines in both serum and liver, especially IL-6, IL-1β, and TNF-α [32,35,71,74,77]. IL-6 is expressed in many inflammatory cells, regulating various biological processes, including inflammation and insulin resistance. The analysis of the applied doses of *Vaccinium* extracts or nutraceuticals indicated a decrease in serum IL-6 levels [32,35,71,74,77,84].

The regulator of the metabolic inflammatory signaling pathway in the liver is NF-κB, which controls the production of various proinflammatory cytokines, including TNF-α and IL-6. Moreover, NF-κB influences liver fibrogenesis by controlling hepatocyte damage, which is a significant factor driving fibrogenic responses in these cells [3,71,72]. Morrison et al. [72] observed that a high-cholesterol diet activated inflammatory signaling pathways (IL-1β, TNF-α, TGF-β) and identified a positive correlation between free cholesterol levels and NF-κB activity, indicating NF-κB as an effector of cholesterol-induced inflammation. Similarly, Faheem et al. [71] found that cranberry supplementation resulted in a significant reduction in hepatic NF-κB expression. Glisan et al. [33] found that cranberry may alleviate liver inflammation by influencing the TLR4/NF-κB signaling pathway and its subsequent effects on TNF-α and the mRNA levels of Cox2 and TNF-α in the liver. The TLR4/NF-κB pathway is essential for regulating the expression of proinflammatory genes, including IL-1β, COX-2, and TNF-α.

According to data analysis, *Vaccinium* extracts administered in various forms improved liver dysfunction by reducing AST and ALT activity, as well as diminishing lipid droplet accumulation and decreasing the expression of FAS and ACC1 in the livers of mice fed a high-fat diet [31,32,78,82]. This indicates their influence on de novo lipogenesis and the potential benefits for NAFLD. Fatty acids can also be synthesized from non-lipid precursors through de novo lipogenesis, a process regulated at the transcriptional level by ACC1 and FAS. SREBP-1c and carbohydrate response element-binding protein primarily mediate this regulation [78].

Studies have shown that high-fat-diet-induced lipid accumulation in the liver is associated with increased expression of SREBP-1c [100,101]. Supplementation with lingonberry, as demonstrated in the studies by Hewage et al. [31,32], reduced the expression of SREBP-1c, decreased plasma lipid levels, and improved hepatic steatosis. Notably, lingonberry supplementation attenuated Notch1 expression in the liver of mice fed a high-fat diet and inhibited Notch1 signaling, lipogenesis, and palmitic-acid-induced lipid accumulation in HepG2 cells. Notch1 signaling stimulates lipid accumulation in the liver by inducing de novo hepatic lipogenesis in NAFLD through the upregulation of transcriptional activation of fatty acid synthesis genes. Inhibiting Notch significantly reduced cellular lipid accumulation by alleviating SREBP-1c-dependent lipogenesis and increasing fatty acid oxidation [31]. Studies by Ren et al. [75] showed that blueberry juice increased PPAR-α levels, reduced SREBP-1c levels, and decreased PNPLA-3 levels. Other studies by Ren et al. [76] indicated that treatment with blueberry juice resulted in high levels of SIRT1 and PPAR-α proteins, while SREBP-1c levels remained low. Cranberry treatment also reduced hepatic SREBP-1c expression in mice receiving a 5% cranberry dose, though this inhibitory effect disappeared after two weeks [84]. Activation of PPAR-α prevents TG synthesis in liver cells by inhibiting SREBP-1c activity, which is known to be associated with hepatic steatosis. PNPLA-3 is linked to lipid accumulation in liver tissues, particularly in the progression of hepatic steatosis [75]. SIRT1 regulates lipid homeostasis through the upregulation of PPAR-α. It is crucial to understand the pathways by which PPAR-α inhibits SREBP-1c and PNPLA-3 activity and how SIRT1 controls the SREBP-1c/PNPLA-3 pathway [76]. Hewage et al. [32] indicated that supplementation with 1% (*w*/*w*) *Vaccinium vitis-idaea* L. could reduce liver lipid accumulation by downregulating SREBP-1c and restoring AMPK. One of the mechanisms of AMPK action involves the phosphorylation of SREBP-1c, which inhibits its proteolytic cleavage and nuclear translocation, thereby suppressing hepatic lipogenesis.

The enzymes DGAT1 and DGAT2, involved in the final step of triacylglycerol synthesis, also play a role in developing hepatic steatosis. Overexpression of DGAT1 is linked to elevated levels of triacylglycerols incorporated into very-low-density lipoproteins (VLDL), whereas DGAT2 is more closely associated with the synthesis of triacylglycerols stored in the cytosol [117]. Lingonberry supplementation reduced the expression of DGAT1 and DGAT2, which may contribute to low hepatic TG content in high-fat-diet-fed mice. Additionally, lingonberry supplementation inhibited the expression of CD36, a fatty acid uptake receptor, potentially leading to reduced hepatic fatty acid uptake in high-fat-diet-fed mice [32].

Cranberry supplementation also prevented the upregulation of genes involved in lipid metabolism, such as monoacylglycerol O-acyltransferase 1 (Mogat1). Mogat1 plays a role in TG metabolism in the liver, fat absorption in the gastrointestinal tract, and the early development of type 2 diabetes, hepatic steatosis, and obesity. As an enzyme that converts monoacylglycerol to diacylglycerol, Mogat1 expression in the liver notably rises in mouse models fed a high-fat diet and is activated by PPAR-γ [80].

Several studies on NAFLD suggest promising potential for using *Vaccinium* species in antifibrotic therapy. Supplementation with Mirtoselect, cranberry, blueberry, and lingonberry may effectively mitigate disease progression [71,72,77,80]. Mirtoselect significantly reduced liver collagen content and decreased the expression of fibrosis-related genes, such as Col1a1 and Acta2. Cranberry demonstrated similar potential by reducing levels of TGF-β, α-SMA, and hydroxyproline, leading to decreased collagen deposition, while lingonberry supplementation inhibited the upregulation of genes associated with liver inflammation and fibrosis. The antifibrotic potential of extracts from lingonberry, cranberry, and blueberry was confirmed by Sergazy et al. [118]. They demonstrated that in rats treated with these extracts, fibrosis was either absent or had a mild, focal character, suggesting that these extracts may effectively inhibit the development of liver fibrosis in cases of liver damage. The authors hypothesized that this improvement was likely due to the antifibrotic effects of the berry extracts, which might be attributed to a reduction in inflammation and oxidative stress. Sun et al. [119] demonstrated that blueberry anthocyanins might offer protective effects against liver fibrosis induced by CCL4. This effect could be attributed to their ability to reduce ROS generation and associated oxidative damage, lower the impact of proinflammatory cytokines, inhibit hepatic stellate cell activity, and decrease the expression of collagen III and α-SMA, while enhancing the expression of MMP-9.

Clinical research on the use of *Vaccinium* species fruits is limited. The studies reviewed focused exclusively on patients with confirmed NAFLD, excluding those with related conditions. The results suggest that cranberry may have beneficial effects in treating NAFLD through various pharmacological actions, including lowering glycemic response, HOMA-IR, lipid levels, and liver enzymes (AST, ALT, ALP) [34,85]. These clinical studies primarily used non-invasive diagnostic methods like ultrasound. Both trials are considered high quality; however, since the review includes only two clinical trials, it is not easy to perform a meta-analysis and, thus, it was decided to forgo this approach. Additionally, the studies cited employed groups of varying and relatively small sample sizes, different doses and methods of cranberry extract administration, and varying study durations. Despite these limitations, the findings underscore the potential of cranberry fruits in NAFLD treatment and highlight the need for further in-depth clinical research to understand their efficacy and fully establish standardized treatment protocols.

## 5. Future Research

Future research on the use of *Vaccinium* plants in the treatment of NAFLD and liver diseases should focus on several key areas. Firstly, it is essential to conduct more clinical studies involving a broad range of patients, including those with comorbid conditions associated with NAFLD, to more accurately assess the effectiveness of these plants in diverse populations. Randomized controlled trials should be designed to assess the efficacy of *Vaccinium* berries and leaves in various forms, such as whole extracts, standardized extracts rich in anthocyanosides, and isolated bioactive compounds. These trials should explore their effects on liver function, lipid profiles, and inflammatory markers over extended periods. Additionally, RCTs should compare different dosages and delivery methods, such as oral supplementation versus dietary inclusion, to determine the most effective approach. Secondly, understanding the molecular mechanisms through which bioactive compounds in *Vaccinium* fruits and leaves influence lipid metabolism and liver inflammation will be crucial. Further studies should also aim to optimize dosages and forms of supplementation to maximize health benefits. An important step is evaluating the long-term effects of these extracts to ensure that no adverse side effects occur and that therapeutic benefits are sustained. It may be interesting to investigate whether high levels of specific anthocyanosides from *Vaccinium* are associated with positive outcomes. However, the focus should be on using modern and standardized methods for the measurement and diagnosis of liver diseases, including NAFLD/NASH. It is necessary to investigate ways to enhance the bioavailability of bioactive compounds from *Vaccinium*. This can be achieved by modifying the delivery form, such as using nanotechnology, microencapsulation, or liposomal formulations, which may improve the absorption and stability of these compounds. Finally, future research should investigate potential interactions between *Vaccinium* supplements and other medications used in NAFLD treatment to ensure safety and efficacy.

In summary, preclinical and clinical studies on extracts, juices, or nutraceuticals derived from *Vaccinium* have demonstrated a favorable safety profile. These findings may be attributed to the small cohort sizes or the use of low doses of *Vaccinium* preparations, which may have fallen below the threshold for biological efficacy. However, it is essential to note that these studies exhibit significant variability in the active compounds used, the matrices administered, the dosages, and the duration of treatment. Nonetheless, the promising results from these studies suggest that more extensive cohort studies could further clarify the efficacy of *Vaccinium*-based interventions in the treatment of liver diseases.

## 6. Strength and Limitations

This review provides a thorough analysis of *Vaccinium* spp. berries in preventing and treating NAFLD, covering clinical, preclinical, and in vitro studies from the past 14 years. It was carried out according to PRISMA guidelines by three independent researchers who evaluated the internal quality of the selected reports. Additionally, applying the Jadad scale and the methodology of the included animal studies following the Stroke Therapy Academic Industry Roundtable in this review provides an objective framework, minimizing subjective biases. Both trials are regarded as high quality according to the Jadad scale. The quality assessment of preclinical studies, according to STAIR, indicates that the included studies received ratings reflecting the fulfillment of fundamental methodological criteria. Results from studies rated at level 4 may suggest potential therapeutic efficacy but require further research to confirm these observations. It may be advisable to supplement the findings with additional analyses or conduct small confirmatory experiments before advancing to the clinical phase. For in vitro studies, interpreting the collective evidence is difficult because of variations in cellular models, experimental protocols, and molecular pathways evaluated.

This study has certain limitations. The first limitation of the study is that the findings in this work are based on animal studies (in vivo), and therefore may not be directly applicable to clinical practice. Secondly, the discussed clinical studies are limited and pertain to only two papers analyzing cranberry capsules administered in various doses. Additionally, the sample sizes were small, with the most significant number of participants only 110. As a result, the obtained data may be considered controversial. Third, the short observation periods present another limitation of the identified studies. All of the clinical studies had a duration of 6 months, meaning the long-term impact of cranberries on treating NAFLD and related diseases remains to be discovered. In animal studies, diverse dietary models and various forms of *Vaccinium* extracts have been employed to investigate NAFLD. Another limitation is that the results were based exclusively on two animal species, namely rats and mice, indicating the need for comparative studies with other species. However, due to NAFLD’s complex and multifaceted nature, only some animal models fully capture the disease’s entirety. Another limitation is that this current review serves as a proof of concept, with the findings in this study laying the groundwork for future clinical trials.

## 7. Conclusions

NAFLD is a common chronic liver condition where the primary therapeutic approach focuses on lifestyle modifications such as dietary changes and physical activity. Preclinical and preliminary clinical studies suggest that extracts from the fruits and leaves of *Vaccinium* plants may have a beneficial impact on the treatment of NAFLD. These extracts may prevent liver steatosis and its progression to NASH by reducing oxidative stress, regulating apoptosis, and decreasing inflammation. *Vaccinium* extracts’ mechanisms include reducing reactive oxygen species production, improving mitochondrial content and membrane potential, and regulating apoptosis-related proteins (Bcl-2, Bax, HO-1). Additionally, these extracts may affect the Nrf-2 pathway, crucial in the antioxidant response. *Vaccinium* extracts exhibit anti-inflammatory effects by lowering levels of proinflammatory cytokines such as IL-6, IL-1β, and TNF-α. They also reduce NF-κB activity, which regulates the production of proinflammatory cytokines and affects liver fibrogenesis. Furthermore, *Vaccinium* extracts influence metabolic processes, including de novo lipogenesis, by decreasing the expression of critical enzymes such as FAS, ACC1, DGAT1, and DGAT2 and regulating SREBP-1c and PPAR-α activity, leading to reduced lipid accumulation in the liver. In summary, extracts from *Vaccinium* plants show therapeutic potential in treating NAFLD by offering benefits through their antioxidant, anti-inflammatory, and lipid-metabolism-modulating effects. Evidence from clinical trials, animal studies, and in vitro models regarding the use of *Vaccinium* species in mitigating NAFLD provides a substantial source of supporting positive evidence that should be considered before completely dismissing the potential therapeutic role of these plants. However, further research is needed to assess their efficacy and safety fully.

## Data Availability

The bibliographic query is stored in the Repository for Open Data https://doi.org/10.18150/4KJRYQ.
